# Surface properties correlate to the digestibility of hydrothermally pretreated lignocellulosic Poaceae biomass feedstocks

**DOI:** 10.1186/s13068-017-0730-3

**Published:** 2017-02-23

**Authors:** Demi T. Djajadi, Aleksander R. Hansen, Anders Jensen, Lisbeth G. Thygesen, Manuel Pinelo, Anne S. Meyer, Henning Jørgensen

**Affiliations:** 1 Department of Chemical and Biochemical Engineering, Technical University of Denmark, Søltofts Plads Building 229, 2800 Kongens Lyngby, Denmark; 2 Department of Plant and Environmental Sciences, University of Copenhagen, Thorvaldsensvej 40, 1871 Kongens Lyngby, Denmark; 3 Department of Geosciences and Natural Resource Management, University of Copenhagen, Rolighedsvej 23, 1958 Frederiksberg C, Denmark

**Keywords:** Hydrothermal pretreatment, Enzymatic hydrolysis, Hemicellulose, Wettability, 2D nuclear magnetic resonance (NMR), Attenuated total reflectance-Fourier transform infrared (ATR-FTIR), Comprehensive microarray polymer profiling (CoMPP), Contact angle measurements

## Abstract

**Background:**

Understanding factors that govern lignocellulosic biomass recalcitrance is a prerequisite for designing efficient 2nd generation biorefining processes. However, the reasons and mechanisms responsible for quantitative differences in enzymatic digestibility of various biomass feedstocks in response to hydrothermal pretreatment at different severities are still not sufficiently understood.

**Results:**

Potentially important lignocellulosic feedstocks for biorefining, corn stover (*Zea mays* subsp. *mays* L.), stalks of *Miscanthus* × *giganteus*, and wheat straw (*Triticum aestivum* L.) were systematically hydrothermally pretreated; each at three different severities of 3.65, 3.83, and 3.97, respectively, and the enzymatic digestibility was assessed. Pretreated samples of *Miscanthus* × *giganteus* stalks were the least digestible among the biomass feedstocks producing ~24 to 66.6% lower glucose yields than the other feedstocks depending on pretreatment severity and enzyme dosage. Bulk biomass composition analyses, 2D nuclear magnetic resonance, and comprehensive microarray polymer profiling were not able to explain the observed differences in recalcitrance among the pretreated feedstocks. However, methods characterizing physical and chemical features of the biomass surfaces, specifically contact angle measurements (wettability) and attenuated total reflectance-Fourier transform infrared (ATR-FTIR) spectroscopy (surface biopolymer composition) produced data correlating pretreatment severity and enzymatic digestibility, and they also revealed differences that correlated to enzymatic glucose yield responses among the three different biomass types.

**Conclusion:**

The study revealed that to a large extent, factors related to physico-chemical surface properties, namely surface wettability as assessed by contact angle measurements and surface content of hemicellulose, lignin, and wax as assessed by ATR-FTIR rather than bulk biomass chemical composition correlated to the recalcitrance of the tested biomass types. The data provide new insight into how hydrothermal pretreatment severity affects surface properties of key Poaceae lignocellulosic biomass and may help design new approaches to overcome biomass recalcitrance.

**Electronic supplementary material:**

The online version of this article (doi:10.1186/s13068-017-0730-3) contains supplementary material, which is available to authorized users.

## Background

Pretreatment is an important process step in the processing of recalcitrant lignocellulosic biomass and is employed to enhance the susceptibility of the biomass to enzymatic deconstruction. Among various pretreatment technologies developed and tested [1–3], hydrothermal pretreatment (HTP) has been employed in recently established demonstration and commercial scale second generation ethanol plants, i.e., the Inbicon demonstration plant in Denmark [4] and the full scale ethanol plant of Beta Renewables in Italy [5]. HTP is based on the treatment of biomass with steam and no addition of a catalyst [4]. The advantages of HTP include that operation without catalyst addition (e.g., acids) enables the use of less expensive alloys for construction of reactors and gives lower operational costs [4]. Common to all pretreatment technologies is that they modify the cell wall structure and composition and thereby make the cellulose more susceptible to enzymatic attack.

HTP results in partial defibrillation and fractionation of the biomass due to solubilization of hemicellulose and redistribution of lignin [6, 7]. The extent of both the hemicellulose solubilization and the lignin redistribution depends on the severity of the treatment (time, temperature, particle size, and mechanical shear imposed on the material). HTP is usually performed in the range of 180–200 °C for 10–20 min, because the treatment severity is a compromise between the intention to amend the cellulose to enzymatic attack and production of cellulase inhibitors that may retard the enzymatic efficacy [4, 7, 8]. During HTP employing the conditions above, water is auto-ionized and acts as a catalyst that hydrolyzes glycosidic bonds in hemicellulose in addition to releasing notably acetic acid from the biomass which further acts to catalyze the depolymerization of hemicellulose [9, 10]. The extent of hemicellulose depolymerization is affected by the intensity of the reaction which is expressed as severity factor (log *R*_0_) [10, 11], whereas the extent of cellulose hydrolysis and solubilization is minor [7, 12]. The lignin on the other hand, depending on its glass transition temperature (*T*_g_), turns into a fluid-like state and during the pretreatment relocates within and on the cell wall material. Redeposited droplets of recondensed lignin are frequently observed on the surface of the pretreated material [6, 13]. This relocation improves accessibility initially due to exposure of a larger cellulose area, but the lignin droplets themselves have been suggested to sterically hinder cellulolytic enzymes attack or act to unproductively bind cellulases [14, 15]. Nevertheless, the removal of hemicellulose and the redistribution of lignin during HTP are thought to render cellulose more susceptible towards enzymatic deconstruction [16–18]. Generally, there is a good correlation between severity, hemicellulose solubilization, and the degree of cellulose depolymerization, i.e., at higher severity more hemicellulose is solubilized and the cellulose hydrolysis is improved [7, 19–21], although profound differences in recalcitrance can occur within even closely related cultivars and botanical parts of the same species, as shown for, e.g., wheat straw [22, 23]. It has nevertheless been common practice to assess the removal of hemicellulose from the original material as indicator of hydrothermal pretreatment effectiveness [7, 19–21].

Recently, differences in accessibility of water to cellulose were found to partly correlate to the accessibility of the enzymes to cellulose and thereby the cellulose convertibility [24–27]. The ability to interact with water, i.e., the wettability, represented as surface hydrophobicity through initial water contact angle measurement, has been found to improve after organosolv and steam explosion pretreatment of wheat straw; therefore suggesting a connection between digestibility and wettability [28]. Despite this recent progress the quantitative aspects of the molecular and structural mechanisms governing biomass recalcitrance of pretreated biomass are not sufficiently understood when it comes to responses to different pretreatment severities and differences across feedstocks.

The objective of this study was to obtain improved knowledge of how the chemistry, physics, and enzymatic digestibility of industrially relevant Poaceae biomass feedstocks respond to different HTP severities and notably to attempt to identify factors that correlate with the recalcitrance of a biomass with a given composition.

## Results and discussion

### Composition

The compositions of the solid fraction of the biomass feedstocks were compared among the different severity levels and with respect to the original untreated (raw) materials on a dry matter (DM) basis (Table 1). When calculated from monomeric composition assessment (using the NREL biomass analysis protocol [29]), the arabino-(galacto-)/xylan contents decreased significantly along with concomitant increase of glucan content as the pretreatment severity increased. This indicated solubilization of hemicellulose which in turn will be expected to expose cellulose and improve enzymatic digestibility [6, 7, 10]. After HTP, the lignin content was also higher than in the untreated biomass, although lignin levels remained relatively stable irrespective of severity factor in *Miscanthus* × *giganteus* stalks (MS) and wheat straw (WS) (Table 1). The lignin content in corn stover (CS) was lower than in the other biomass feedstocks and remained the same even after pretreatment. The carbohydrate contents of all pretreated biomass feedstocks were relatively similar among corresponding severity factors except for xylan in MS, which was lower than in CS and WS.

**Table 1:** Composition of untreated (raw) and hydrothermally pretreated (severity factor: log *R*_0_) biomass feedstocks

Biomass—log *R*_0_	Arabinan	Galactan	Glucan	Xylan	Mannan	Lignin^1^	Ash	Extractives
	(% w/w DM)							
Raw CS	*3.4* ± *0.1* ^a^	*1.4* ± *0.1* ^a^	*43.7* ± *0.7* ^c^	*23.8* ± *0.1* ^a^	*0.5* *±* *0.0* ^a^	*19.4* ± *1.3* ^c^	*2.3* ± *0.1*	*6.7* *±* *0.6*
CS—3.65	0.7 ± 0.1^d^	0.4 ± 0.0^c^	55.5 ± 3.1^ab^	14.7 ± 0.8^c^	0.2 ± 0.0^c^	23.8 ± 2.3^c^	4.1 ± 0.5	
CS—3.83	0.4 ± 0.0^e^	0.2 ± 0.0^efg^	55.7 ± 1.3^ab^	11.2 ± 0.5^de^	0.1 ± 0.0^d^	22.4 ± 0.8^c^	4.6 ± 1.2	
CS—3.97	0.2 ± 0.0^fg^	0.1 ± 0.0 ^g^	61.2 ± 1.1^a^	6.4 ± 0.1^g^	0.1 ± 0.0^d^	19.9 ± 3.9^c^	3.4 ± 0.2	
Raw MS	*3.0* *±* *0.1* ^b^	*0.9* *±* *0.0* ^b^	*38.6* *±* *0.2* ^c^	*19.9* *±* *0.4* ^b^	*0.3* *±* *0.0* ^b^	*23.2* *±* *0.2* ^c^	*2.2* *±* *0.1*	*8.6* *±* *0.3*
MS—3.65	0.6 ± 0.0^d^	0.3 ± 0.0 ^cd^	53.6 ± 2.6^b^	11.3 ± 0.4^d^	0.3 ± 0.0^b^	32.5 ± 2.1^ab^	1.1 ± 0.0	
MS—3.83	0.4 ± 0.0^e^	0.2 ± 0.0^de^	54.7 ± 2.8^b^	7.8 ± 0.6^f^	0.3 ± 0.0^b^	32.2 ± 0.5^ab^	1.5 ± 0.0	
MS—3.97	0.2 ± 0.0^fg^	0.2 ± 0.0^ef^	55.9 ± 2.1^ab^	4.5 ± 0.2 ^h^	0.3 ± 0.0^b^	35.6 ± 0.3^a^	1.6 ± 0.0	
Raw WS	*2.8* *±* *0.0* ^c^	*0.9* *±* *0.0* ^b^	*41.3* *±* *0.2* ^c^	*24.9* *±* *0.7* ^a^	*0.5* *±* *0.0* ^a^	*21.4* *±* *0.2* ^c^	*1.3* *±* *0.0*	*10.6* *±* *0.3*
WS—3.65	0.7 ± 0.0^d^	0.3 ± 0.0 ^cd^	54.8 ± 0.6^ab^	14.7 ± 0.0^c^	0.5 ± 0.0^a^	29.3 ± 0.7^b^	1.4 ± 0.1	
WS—3.83	0.3 ± 0.0^f^	0.2 ± 0.0^def^	58.2 ± 4.7^ab^	9.8 ± 0.4^e^	0.5 ± 0.0^a^	30.8 ± 0.7^b^	1.1 ± 0.2	
WS—3.97	0.1 ± 0.0 ^g^	0.1 ± 0.0 ^fg^	61.2 ± 2.5^a^	6.5 ± 0.2 ^g^	0.5 ± 0.0^a^	30.3 ± 1.1^b^	1.0 ± 0.1	

Data in italics are for untreated (raw) biomass samples

Results are average and standard deviation of triplicate measurements

Different letters indicate significant statistical difference based on ANOVA (*P* ≤ 0.05)

CS, corn stover; MS, *Miscanthus* × *giganteus* stalks; WS, wheat straw

^1^Based on acid insoluble lignin (AIL) and acid soluble lignin (ASL) contents

**Fig. 1:**
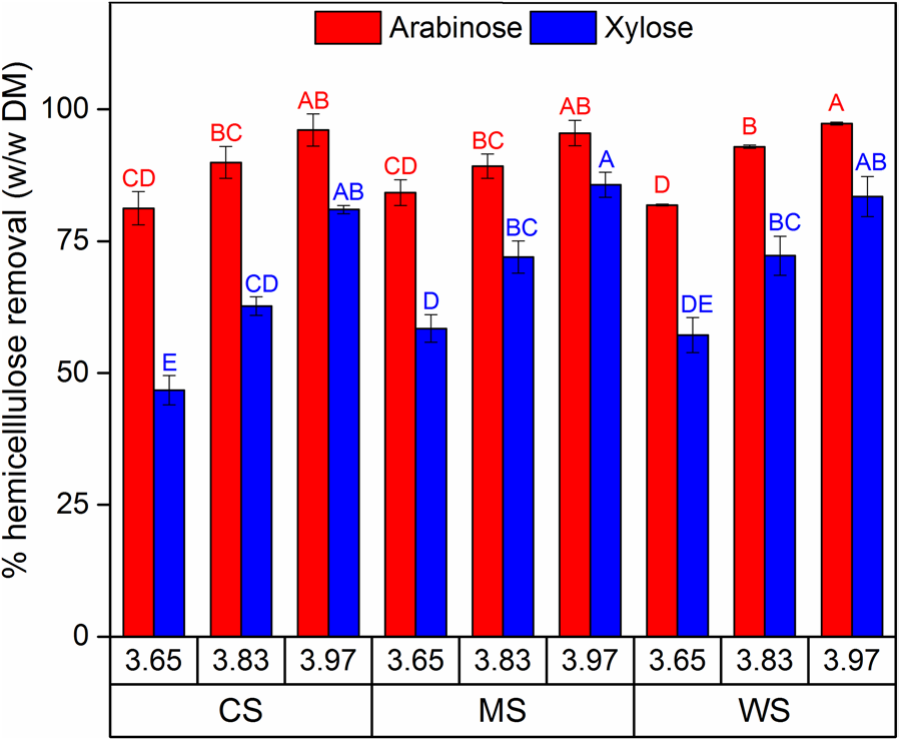
Removal of arabinose (*red*) and xylose (*blue*) relative to the untreated biomass for corn stover (CS), *Miscanthus* × *giganteus* stalks (MS), and wheat straw (WS) at different severity factors (log *R*_0_). Data points represent average ± standard deviation from three technical replicates. *Different letters* indicate significant statistical difference based on ANOVA (*p* ≤ 0.05)

As mentioned, the extent of hemicellulose removal relative to the original raw material has been shown to correlate with digestibility [7, 20, 21] and cellulose accessibility [30] of pretreated lignocellulosic biomass materials. The results indicated sizeable removal of hemicellulose, assessed as degree of arabinose and xylose removal, in response to the applied severity factor, i.e., increased hemicellulose removal at elevated severity factor (Fig. 1). However, the extent of hemicellulose removal was similar across all three biomass feedstocks (Fig. 1). The extent of xylose removal was similar to that reported previously for corn stover [19] and wheat straw [31, 32] given the same range of severity factor.

### Enzymatic degradation

**Fig. 2:**
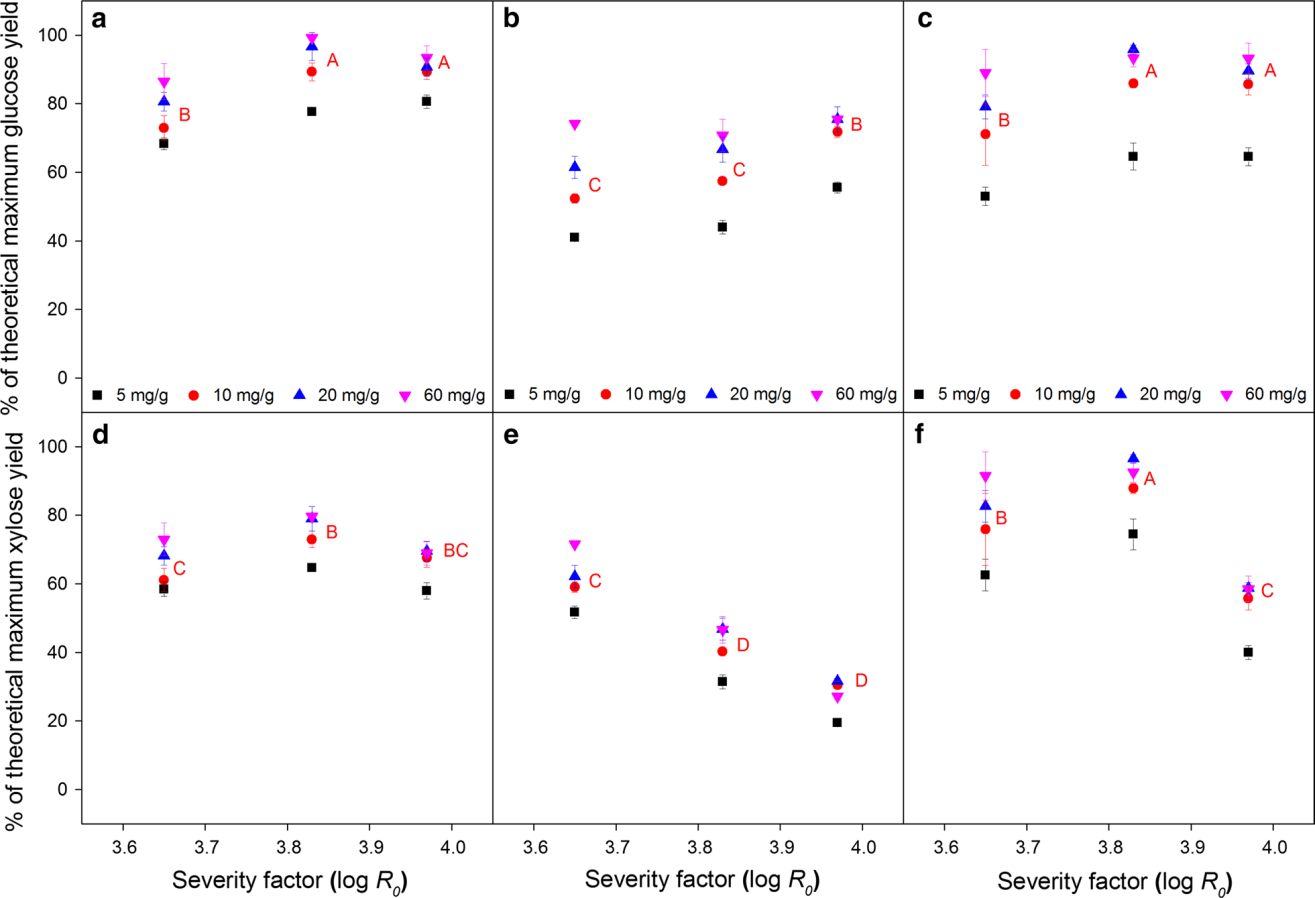
Glucose (**a**–**c**) and xylose (**d**–**f**) release after 72 h enzymatic hydrolysis of hydrothermally pretreated (**a**, **d**) corn stover (CS), (**b**, **e**) *Miscanthus* × *giganteus* stalks (MS), and (**c**, **f**) wheat straw (WS) at different severity factors (log *R*_0_) and enzyme dosages (mg/g). *Data points* represent average and standard deviation from three experimental replicates. *Different letters* indicate significant statistical difference based on ANOVA (*p* ≤ 0.05) for 10 mg/g enzyme dosage series

The digestibility of the three types of biomass feedstock differed in terms of glucose release after enzymatic cellulase treatment. Corn stover (CS) and wheat straw (WS) were more digestible than *Miscanthus* × *giganteus* stalks (MS) as shown by a glucose release of up to 93% of maximum theoretical at the highest severity (log *R*_0_ = 3.97) and enzyme dosage (60 mg protein/g biomass) tested. The corresponding value for MS was 75% (Fig. 2a–c). At other corresponding severities and enzyme dosages, the extents of glucose release from MS were also consistently lower compared to CS and WS. The three biomass feedstocks also responded differently to pretreatment severity and enzyme dosage. CS and WS were less affected by pretreatment severity as the glucose release seemed to be leveling off at log *R*_0_ = 3.83 and 3.97; whereas in the case of MS, there was a slight tendency of increase. In terms of enzyme dosage, CS was the least responsive of the three biomass feedstocks. For CS, the increment of glucose release when increasing enzyme dosage from 5 to 60 mg protein/g DM biomass varied from 16 to 28% across the three severity levels. For MS and WS, the increments were 36–81 and 44–68%, respectively (Fig. 2a–c). Altogether, the results for glucose release revealed that MS is more recalcitrant than CS and WS. MS therefore needs higher severity pretreatment and higher enzyme dosage to obtain high cellulose conversion.

Except for corn stover, the xylose release from the pretreated biomass feedstocks tended to decrease as pretreatment severity increased, but not consistently statistically significant (Fig. 2d–f). This trend of xylose release has been observed previously for both corn stover [33] and ensiled wheat straw [31] hydrothermally pretreated at different severities. In the case of MS, the extent of xylose release was lower than for CS and WS, particularly evident at the higher severities log *R*_0_ > 3.65, where the extent of xylose release achieved with the highest enzyme dosage (60 mg protein/g DM biomass) was lower than the corresponding value for CS and WS with the lowest dosage (5 mg protein/g DM biomass) (Fig. 2d–f).

To our knowledge, the apparent higher recalcitrance of MS compared to CS and WS after HTP has not been reported previously, partly due to limited number of studies comparing all three biomasses. One study comparing total hemicellulose removal during combined steam explosion and dilute sulfuric acid pretreatment found that the obtained yield for MS was slightly lower compared to wheat straw [34]. Furthermore, it took higher severity level for MS to reach the maximal yield as opposed to wheat straw [34]. However, since variation among genotypes/cultivars of *Miscanthus* [35] and wheat [22], which affects the release of sugars after HTP and enzymatic hydrolysis of the stalks/straws has been reported, conclusions on their inherent species-related recalcitrance are not necessarily universal. The increment of xylose release (Fig. 2d–f) with increased enzyme dosage (from 5 up to 60 mg protein/g DM biomass) for each severity level varied less (19–25, 39–63, and 24–47% for CS, MS, and WS, respectively) than the corresponding glucose release (Fig. 2a–c). The data indicate that the hemicellulose remaining after increased severity pretreatment was less susceptible to the enzymes. One possible factor limiting the susceptibility of xylan to enzymes is the degree of arabinose substitution, but acetylation and diferulate cross-links may also hinder the action of xylanolytic enzymes [36]. The substitutions can persist even after pretreatment; pointing to the fact that addition of accessory enzymes such as acetyl xylan esterase [37] and arabinofuranosidases [38] may be needed to further improve the enzymatic hydrolysis. 2D NMR and CoMPP were performed to investigate the decorations of hemicellulose in the biomasses.

## 2D Nuclear magnetic resonance (NMR)

^13^C-^1^H HSQC (Heteronuclear single quantum coherence) spectra (Additional file 1: Figures S1–S12) were used for data analysis. The lowest contour peak integration values showed substantial reduction of acetylated positions (2-*O*-Ac-β-d-Xyl*p* and 3-*O*-Ac-β-d-Xyl*p*) in the fiber fraction of biomasses as a result of HTP. The values decreased as the pretreatment severity increased (Table 2). This is in accordance with previous observations showing that acetylated groups are cleaved during HTP [10, 39]. The results also indicated that acetyl groups once belonging to the hemicellulose moieties of MS and WS were removed substantially to the same extent. However, the values for CS were not able to be processed since the phenylcoumaran structure (Additional file 1: Figure S13) used as reference was not present [40] (Additional file 1: Figures S1–S4). For WS, a substantial reduction of uronic acid (4-*O*-methyl glucuronosyl) was observed after HTP and increased severity (Table 2). Even though the presence of 4-*O*-methyl glucuronosyl residues in arabinoxylans is known to be prominent in the vegetative parts of grasses [41], it was not detected for MS.

**Table 2:** ^13^C-^1^H HSQC NMR contour integration values for acetylated xylosyl and uronic acid relative to phenylcoumaran-α

Structure	MS				WS			
	Raw	3.65	3.83	3.97	Raw	3.65	3.83	3.97
Phenylcoumaran-α	1	1	1	1	1	1	1	1
2-*O*-Ac-β-d-Xyl*p*	38.38	9.99	5.45	3.60	31.26	8.74	4.92	2.38
3-*O*-Ac-β-d-Xyl*p*	20.76	9.20	5.42	3.10	27.56	10.36	5.41	2.58
4-*O*-MeGlcA	^a^	^a^	^a^	^a^	4.32	1.40	0.73	0.07

2-*O*-Ac-β-d-Xyl*p* and 3-*O*-Ac-β-d-Xyl*p*: acetylated xylosyl

4-O-MeGlcA: uronic acid

^a^Peaks were too small for accurate determination

### Comprehensive microarray polymer profiling (CoMPP)

**Fig. 3:**
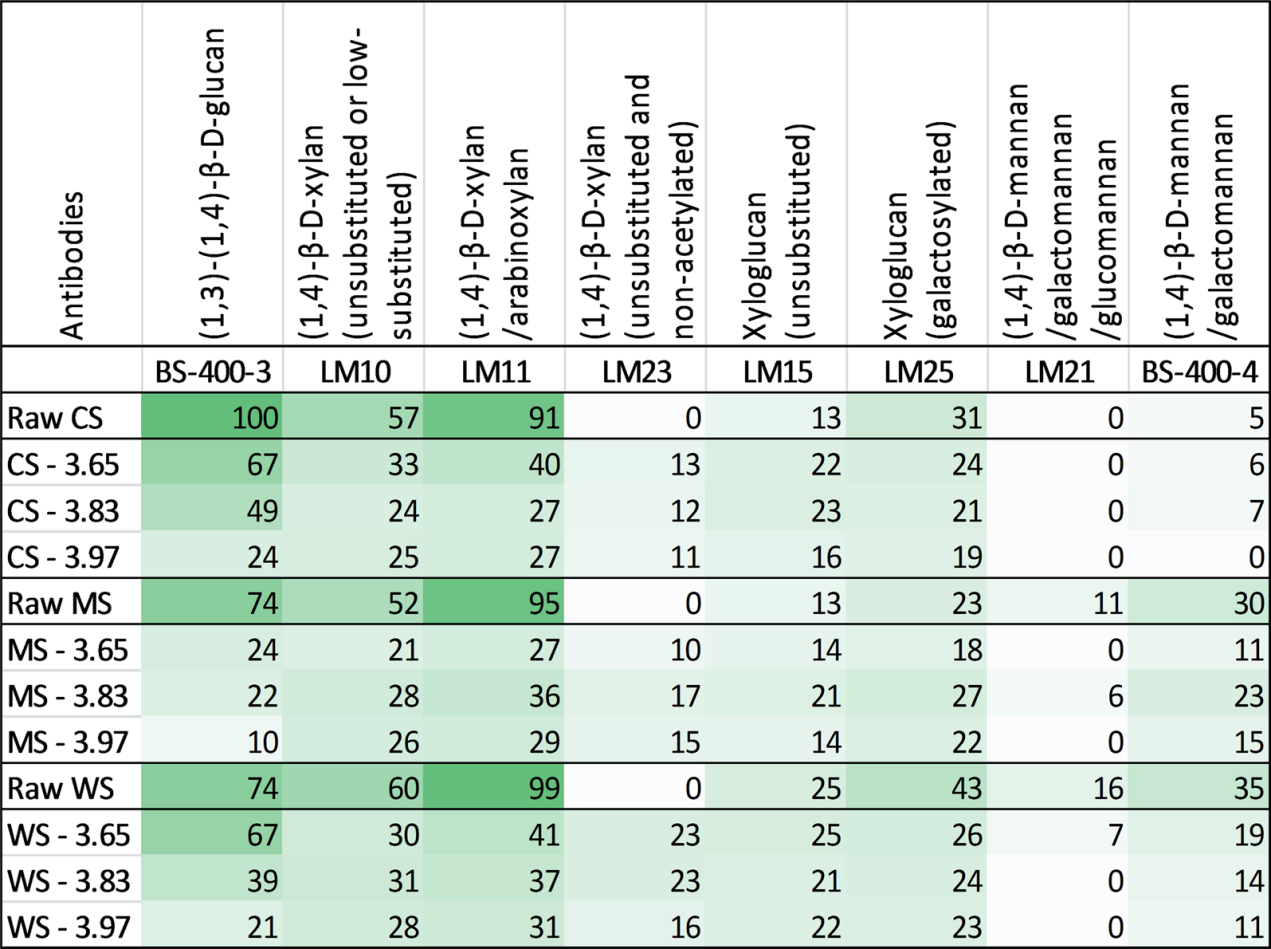
CoMPP results for untreated (raw) and hydrothermally pretreated corn stover (CS), *Miscanthus* × *giganteus* stalks (MS), and wheat straw (WS) at different severity factors (log *R*_0_) after extraction with CDTA and 4 M NaOH in 0.1% (w/v) NaBH_4_

CoMPP analysis was performed to infer whether there are particular structural polysaccharides that are more preferentially removed in some biomasses compared to others, thus possibly explaining the contribution towards recalcitrance. Based on several models of cell walls in grasses [42–44], the cellulose microfibrils can be covered and/or tethered by structures like arabinoxylan (AX), mixed linked glucan (MLG), and xyloglucan (XG). The signals derived from antibodies binding to MLG, xylan, and AX (LM10 and LM11) had high intensity in all of the untreated biomass and they dropped significantly after pretreatment (Fig. 3). The effect seemed to correlate with the applied severity and the observed hemicellulose removal (Fig. 1). LM10 is known to preferably bind to unsubstituted or low-substituted xylans, whereas LM11 binds to arabinoxylan as well as unsubstituted xylan [45, 46]. This is in accordance with the observed significant removal of arabinose and xylose after HTP (Fig. 1). The signals derived from binding of LM23, on the other hand, were increased after HTP for all biomass feedstocks (Fig. 3). Since LM23 is known to bind to unsubstituted and non-acetylated xylans [47], the increase of signals can be expected to happen due to removal of acetyl groups. This is in accordance with the 2D NMR observation of decreased acetylation (Table 2).

The signals derived from antibodies binding to xyloglucans (LM15 and LM25) and mannans (LM21 and BS-400-4) remained relatively stable after HTP, which was in agreement with the mannan content (Table 1). In general, the findings are also in agreement with previous results on wheat straw [44] in which XG and mannans were suggested to be bound more tightly to the microfibrils in the cell wall matrix. On the contrary, MLG and AX were found to be rather loosely bound in the cell wall matrix, shielding the cellulose microfibrils from enzymatic attack until released after pretreatment [44, 48]. The CoMPP results thus revealed that these changes in plant cell wall structural polysaccharides composition, which was previously observed only in WS [44, 48], also applied to CS and MS. Regardless of this, the quantitative composition data coupled with the CoMPP and 2D NMR indicated that hemicellulose decoration or substitution was likely not the factor conferring higher recalcitrance in MS compared to CS and WS.

### Wettability of biomass

**Fig. 4:**
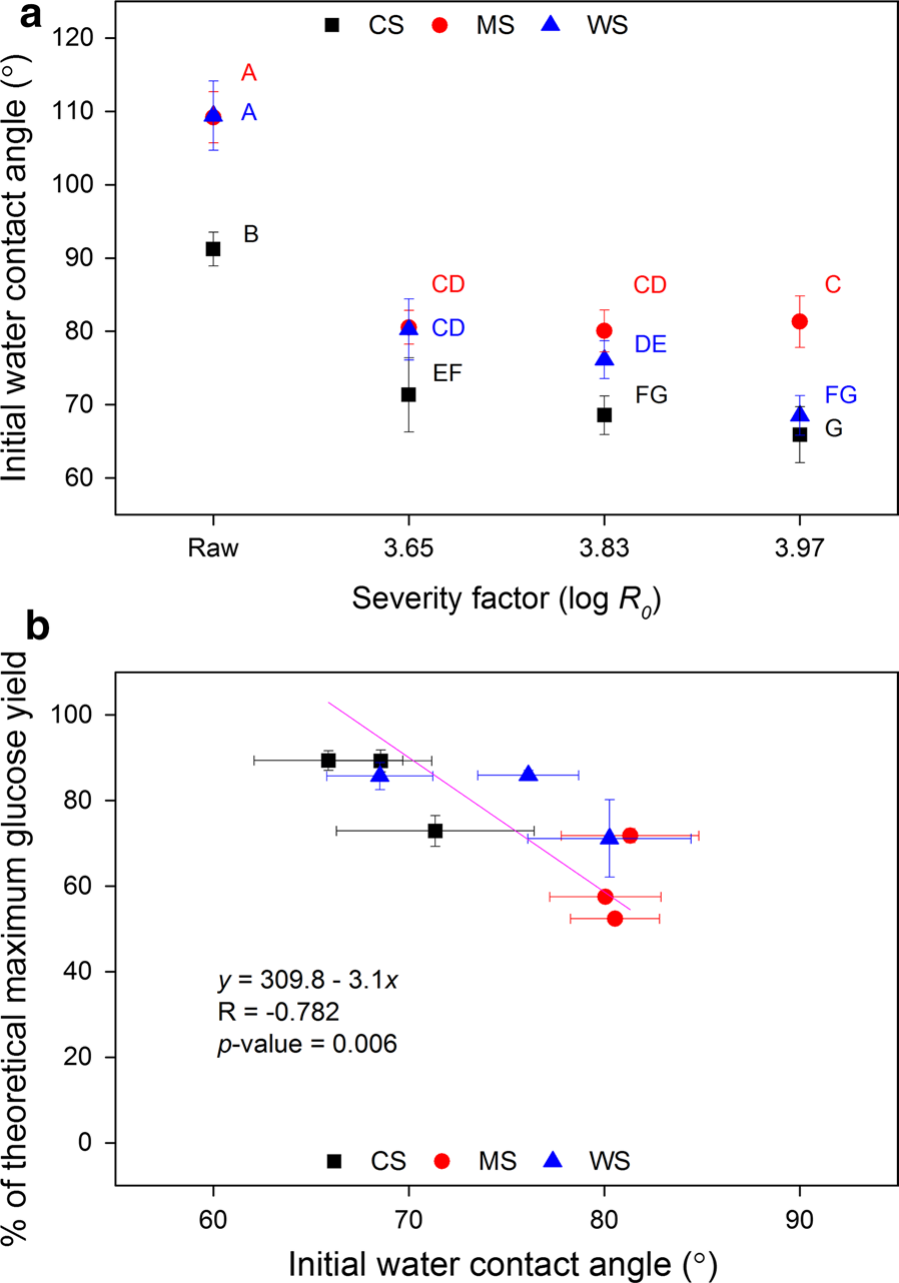
Initial water contact angle (**a**) of raw and hydrothermally pretreated corn stover (CS), *Miscanthus* × *giganteus* stalks (MS), and wheat straw (WS) at different severity factors (log *R*_0_). *Data points* represent average and standard deviation from five technical replicates. *Different letters* indicate significant statistical difference based on ANOVA (*p* ≤ 0.05). Scatter plot (**b**) of glucose release after 72 h enzymatic hydrolysis at 10 mg/g dosage for pretreated CS, MS, and WS at three severity factors with corresponding initial water contact angle prior to hydrolysis. The strength of linear relationship between paired data is indicated by Pearson’s correlation coefficient (*R*) and *t* test of the regression slope (significant if *p* < 0.05)

Since even detailed chemical assessment of the bulk biomass did not fully explain the observed differences in enzymatic digestibility in response to different pretreatment severities and notably between the different feedstocks, we hypothesized that surface hydrophobicity might play a role, and in turn that assessment of physical properties of the biomass surface might provide quantitative clues to explain the observed differences in biomass digestibility. Interaction between biomass and water as shown using NMR analysis [49] and water retention value [25, 26] has been found to correlate well with cellulose accessibility, adsorption of cellulases, and biomass digestibility. Biomass–water interaction can also be evaluated using water contact angle measurement (CAM), which depicts the wettability of the solids’ surface through relative measurement of surface hydrophobicity. The higher the contact angle, the more hydrophobic the surface of the material is, and the lower wettability it has [50, 51]. Measurement of the initial (instantaneous) water contact angle of milled biomass particles pressed into a tablet showed significantly lower initial water contact angles of the hydrothermally pretreated biomass materials compared to the raw materials for all three Poaceae biomasses (Fig. 4a). This is in accordance with previous studies that found the reduction of initial water contact angle after steam explosion and organosolv treatment of wheat straw [28], after autohydrolysis of poplar wood chips [52], as well as after chemical and enzymatic treatments of wheat straw [53].

In general, the initial water contact angle had a negative correlation (*R* = −0.782; *p* = 0.006) with the glucose release after 72 h at 10 mg protein/g DM biomass dosage (Fig. 4b). However, this negative correlation was more pronounced for CS and WS than for MS (the contact angle values for MS after HTP varied less in response to increased pretreatment severity than those for CS and WS) (Fig. 4a, b). Furthermore, the initial water contact angle values after HTP were lower for CS and WS compared to the MS values (Fig. 4a), which correlated negatively to the overall observed higher glucose (Fig. 2a–c) and xylose (Fig. 2d–f) release across severities for CS and WS compared to MS. Wettability assessment using contact angle measurement has been shown previously to correlate negatively with hydrolysis rate and digestibility of pure cellulose [51, 54] as well as adsorption of cellulases onto pretreated wheat straw samples [28]. The results obtained here appear to distinguish MS from the other biomass feedstocks CS and WS. As the initial water contact angle assesses the chemical and/or physical properties of the surface of biomass materials, the data suggest that differences in surface properties of pretreated *Miscanthus* × *giganteus* stalks rather than bulk chemical composition traits play a role in the observed higher recalcitrance of MS when compared to the recalcitrance of CS and WS. Additionally, these observations also indicated that assessment of surface wettability may be able to predict biomass digestibility across different hydrothermally pretreated feedstocks.

### Attenuated total reflectance-Fourier transform infrared (ATR-FTIR) spectroscopy

**Fig. 5:**
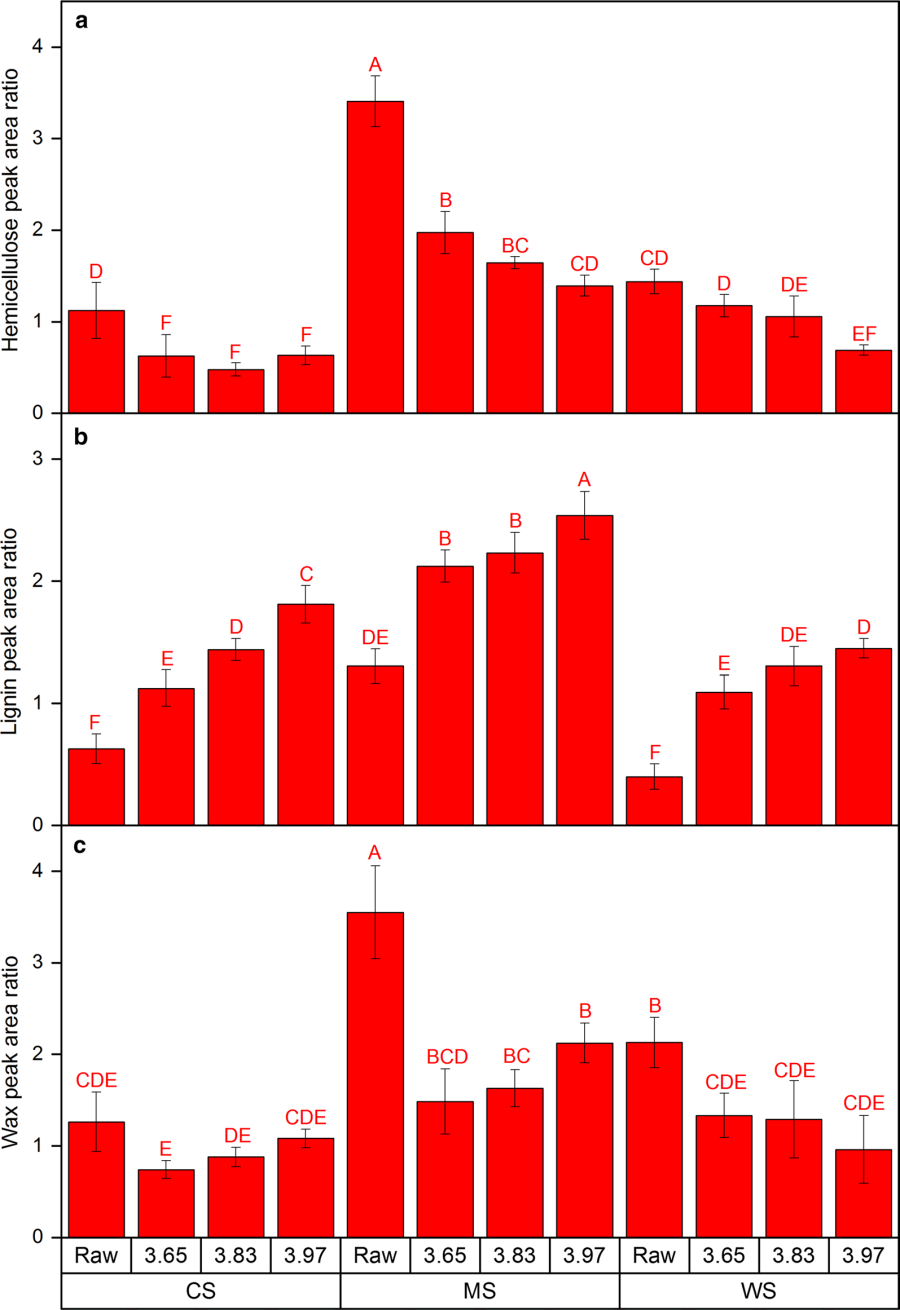
ATR-FTIR peak area ratio of wavenumbers representing **a** hemicellulose (1732 cm^−1^), **b** lignin (1508 cm^−1^), and **c** wax (2918 cm^−1^) each relative to that of holocellulose (895 cm^−1^) for raw and hydrothermally pretreated corn stover (CS), *Miscanthus* × *giganteus* stalks (MS), and wheat straw (WS) at different severity factors (log *R*_0_). *Data points* represent average and standard deviation from five technical replicates. *Different letters* indicate significant statistical difference based on ANOVA (*p* ≤ 0.05)

Similar to the CAM, ATR-FTIR reflects the chemical composition of the surface (estimated penetration depth of 0.57–1.85 μm) of the milled biomass samples. Therefore, it is likely that ATR-FTIR data (Additional file 1: Figures S14–S16) can reveal information about chemical and/or physical features causing the change in observed wettability and hence digestibility. The ATR-FTIR results revealed significant differences that again distinguished MS significantly from CS and WS. Based on the hemicellulose/holocellulose peak area ratio (1732/895 cm^−1^) (Fig. 5a), MS initially had the highest apparent surface abundance of hemicellulose relative to holocellulose (ASA-H/C) compared to the corresponding untreated (raw) CS and WS samples. After HTP, the ASA-H/C levels decreased as the severity increased for all three Poaceae biomass feedstocks; a result which is in line with the hemicellulose (arabinose and xylose) removal observed through composition analysis (Fig. 1). However, for each pretreatment severity level, the values of ASA-H/C were consistently higher in MS compared to CS and WS (Fig. 5a). CS had the lowest ASA-H/C among the three feedstocks, which might imply why it had higher overall digestibility (glucose release) relative to the others (Fig. 2a–c). This difference among the materials was not apparent based on the composition analysis, which yielded similar hemicellulose content for all three biomass feedstocks at each corresponding severity factor (Table 1). Accordingly, the digestibility of biomass correlated strongly and negatively to ASA-H/C; a correlation which was not evident from bulk hemicellulose (arabino-/xylan) composition or hemicellulose (arabinose and xylose) removal after pretreatment (Table 3; Additional file 1: Figures S17, S21, S23).

**Table 3:** Linear regression parameters of surface and bulk composition with digestibility and wettability of pretreated biomass

Parameters		% of theoretical maximum glucose yield^a^				Initial water contact angle (°)			
		*t* test of regression^b^		Pearson’s correlation		*t* test of regression^b^		Pearson’s correlation	
		*p* value	Trend	*R*	Trend	*p* value	Trend	*R*	Trend
Surface^c^	Hemicellulose	<0.001	Strong	−0.883	Strong negative relationship	0.003	Strong	0.876	Strong positive relationship
	Lignin	0.034	Weak	−0.477	Possible negative relationship	0.076	None	0.431	No significant relationship
	Wax	0.081	None	−0.521	Possible negative relationship	0.007	Strong	0.801	Strong positive relationship
Bulk^d^	Cellulose	0.025	Weak	0.760	Strong positive relationship	0.018	Weak	−0.723	Strong negative relationship
	Hemicellulose	0.086	None	−0.215	No significant relationship	0.652	None	0.104	No significant relationship
	Lignin	0.044	Weak	−0.586	Possible negative relationship	0.008	Strong	0.829	Strong positive relationship
	Hemicellulose removal	0.072	None	0.347	No significant relationship	0.427	None	−0.136	No significant relationship

^a^Based on glucose release after 72 h enzymatic hydrolysis at 10 mg/g dosage

^b^ *t* test of the regression slope is significant if *p* value <0.05

^c^Based on ATR-FTIR data (peak area ratio relative to holocellulose)

^d^Based on composition analysis data (% w/w DM) for glucan (cellulose), arabino-/xylan (hemicellulose), lignin (AIL and ASL), and arabinose + xylose (hemicellulose) removal

It is also noteworthy that the surface hemicellulose content (ASA-H/C) had a strong positive correlation with the initial water contact angle (negative with respect to wettability) (Table 3; Additional file 1: Figure S24). This indicated that hemicellulose plays a role in preventing the enzymatic cellulose depolymerization. This can be either by shielding cellulose for enzymatic attack via sterical hindrance or indirectly using other mechanisms. However, the correlation with wettability was not evident when bulk hemicellulose (arabino-/xylan) composition and hemicellulose (arabinose and xylose) removal were considered (Table 3; Additional file 1: Figures S28, S30). Treatment of wheat straw with sodium hydroxide and xylanase which removed hemicellulose was previously reported to reduce the water contact angle, but the correlation to enzymatic cellulose digestibility was not examined [53]. In this study, cellulose digestibility increased after HTP in response to increased severity (Fig. 2a–c) and had negative correlation with the initial water contact angle (positive with respect to wettability) (Fig. 4b). Accordingly, both cellulose digestibility (glucose release) and initial water contact angle (wettability) correlated well with bulk cellulose (glucan) content (Table 3; Additional file 1: Figures S20, S27).

The hydrothermal pretreatment preferentially removes the highly branched hydrophilic hemicellulose moieties thus presumably contributing to reduced digestibility due to reduction of hydrophilicity [55]. It is thus tempting to infer that the residual hemicellulose may not be hydrophilic and actually may adhere strongly to cellulose. In this study, composition analysis (Fig. 1), NMR (Table 2) and CoMPP (Fig. 3) showed major removal of hemicellulose substitutions. Accordingly, hemicellulose with less substitution adsorbed stronger to cellulose compared to more substituted hemicellulose which further provides recalcitrance [56, 57]. Therefore, semi-quantitative assessment of surface hemicellulose and wettability may predict recalcitrance of pretreated biomass materials. Based on the lignin/holocellulose peak area ratio (1508/895 cm^−1^), the apparent surface abundance of lignin relative to holocellulose (ASA-L/C) showed a general trend of increase after HTP in response to increasing severity factor (Fig. 5b). Regardless of this, when comparing the data across all three biomasses, both bulk and surface lignin only had a weak possible negative correlation (*R* ≈ −0.5; *p* ≈ 0.05) with digestibility (Table 3; Additional file 1: Figures S18, S22). It is important to note, however, that unlike the significant increase in ASA-L/C (Fig. 5b), the bulk lignin content for each biomass did not vary across the HTP severity levels (Table 1).

The deposition of lignin in the surface of biomass after dilute acid and hydrothermal pretreatment as seen by microscopic observations [6, 13, 14] was indeed thought to improve enzymatic hydrolysis by providing access to cellulose microfibrils [6, 13]. Later work, however, indicated that during the later stages of enzymatic hydrolysis of hydrothermally pretreated biomass, the lignin droplets accumulated and retarded the glucose release [58]. This is especially true since the droplets have also been found to be inhibitory towards enzymatic hydrolysis [14, 15]. Furthermore, at higher pretreatment severity, the extent of lignin redistribution can be more pronounced [59]. The data obtained thus indicated that within each individual type of biomass, the redistribution of lignin after hydrothermal pretreatment *increased* the lignin abundance at the surface (ASA-L/C) of each biomass with increased pretreatment severity (Fig. 5b). This increased ASA-L/C correlated *positively* to increased glucose release as the pretreatment severity was increased for each biomass (Fig. 2a–c). In contrast, when assessing the ASA-L/C versus glucose release across all three biomasses and across all pretreatments, the correlation changed to become *negative*, and the MS data dominated the model because the overall low glucose release was connected to the highest ASA-L/C values in the dataset (Fig. 5b). Taken together with the data for hemicellulose (Table 3) and contemplating the available knowledge [6, 13, 14, 58, 59], this paradox, i.e., that lignin abundance at the biomass surface (ASA-L/C) correlated *positively* to cellulose digestibility (and pretreatment severity) when each of the biomasses were assessed individually (Fig. 5b), but *negatively* when all three biomasses were compared (across all three severities) (Table 3), led to a suggestion that the positive correlation found between ASA-L/C and cellulose digestibility for the individual biomasses in this study is an artifact. This artifact may be a result of the elevated ASA-L/C with increased pretreatment severity being accompanied by a presumed more significant improvement in cellulose digestibility resulting directly from the effect of hydrothermal pretreatment on the biomass material with increased pretreatment severity in each individual biomass.

Weighing the effect of lignin towards inhibition of cellulose hydrolysis has been difficult to measure since it both obstructs the enzymes and non-productively adsorbs them which has led to different interpretations [16–18]. Studying the inhibitory effect of lignin usually requires an isolation step, which can change the properties of lignin, and this also moves the investigation away from the actual plant cell wall surface, thus removing relevant structural and spatial arrangements. Moreover, the inhibitory effect of isolated lignin to cellulases can also depend on biomass. In softwood (spruce), lignin was reported to be inhibitory [60], whereas in grasses (corn stover and wheat straw) no inhibitory effect was reported [61]. Furthermore the role of lignin as steric hindrance is also known to be affected by the presence of hemicellulose since the direct relationship between lignin redistribution/removal and biomass digestibility was only present when it was coupled to hemicellulose removal [62–64].

Concerning wettability, bulk lignin had a strong positive relation with initial water contact angle (negative with respect to wettability), whereas the surface lignin content did not have it (Table 3; Additional file 1: Figures S25, S29). The bulk composition represents physical existence of lignin, which is generally regarded as hydrophobic. Thus, samples with higher lignin content will have higher initial contact angle as previously reported [28]. However, it is worth noticing again that the bulk lignin content for each biomass did not vary across the HTP severity levels (Table 1), whereas there was a constant trend of increase in ASA-L/C (Fig. 5b). As the ASA-L/C increased, the wettability decreased (increased initial water contact angle) for CS and WS, whereas the wettability remained constant in MS along with increased ASA-L/C (Fig. 4a). This indicates that the relationship between surface lignin and wettability can potentially be strongly negative in some biomass, but not the others. MS indeed had higher ASA-L/C compared to CS and WS already when it had not been pretreated and it increased to even higher extent than the others for each corresponding severity levels.

ATR-FTIR data therefore revealed higher extent of both the original lignin surface distribution and the lignin redistribution (or rather aptly termed resurfacing) after HTP in MS compared to CS and WS which was not apparent using bulk lignin content (Table 1). This correlated negatively with the overall observed lower glucose (Fig. 2a–c) and xylose (Fig. 2d–f) release in MS compared to CS and WS after enzymatic hydrolysis. Investigating the effect of lignin towards wettability might require separate focused studies since other components in biomass can also affect the overall surface properties. Using simulation with molecular theory of solvation, it was shown that lignin and hemicellulose form supramolecular assembly with hydrophobic interaction, which covers cellulose microfibril and expels water from it, thus contributing to recalcitrance altogether [65]. Another simulation study with molecular probes also showed the access of water to cavities in plant cell wall matrix improved after hemicellulose is hydrolyzed and separated from lignin [66].

The wax content has been rarely highlighted in published reports even though it has been considered as one of the structural factors that contributes to lignocellulosic biomass recalcitrance [67]. Correspondingly, wax removal using supercritical CO_2_ on *Miscanthus* × *giganteus* stalks [68] as well as hydrothermal pretreatment [6] and plasma-assisted pretreatment [69] on wheat straw have been shown to correlate with improved hydrolysis yield. Based on the wax/holocellulose peak area ratio (2918/895 cm^−1^), the apparent surface abundance of wax relative to holocellulose (ASA-W/C) decreased significantly after pretreatment, but the level remained relatively stable irrespective of severity (Fig. 5c). Even though it had no strong correlation with digestibility (Table 3; Additional file 1: Figure S19) as with other ATR-FTIR data it also displayed differences among the tested feedstocks. In general, MS had higher ASA-W/C compared to CS and WS for each corresponding severity level. These findings correlated with the overall glucose (Fig. 2a–c) and xylose (Fig. 2d–f) release, which showed that MS was the least digestible. The ASA-W/C had strong positive correlation with the wettability (Table 3; Additional file 1: Figure S26) which can be expected due to its hydrophobic nature and the observation of its removal after extraction [28]. Nevertheless, it was not the only factor affecting wettability after pretreatment since the trend of increase in wettability (decrease of initial water contact angle) was also observed in the materials that were previously solvent-extracted to remove wax [28].

Altogether, investigation of surface properties using ATR-FTIR and contact angle measurement showed that the wettability and hence the observed recalcitrance of biomass are the result of different multi-component interactions on the surface. Even though strong correlation across pretreated biomass feedstocks was seen in connection to surface hemicellulose, overall collective forces from other components, i.e., lignin and wax also pinpointed clues to the particular observed recalcitrance of *Miscanthus* × *giganteus* stalks used in this study. In order to fully understand the changes, future studies need to be directed to investigate the distribution of the different biomass components with respect to ultrastructural architecture of plant cell wall and to take into account changes in physical and/or structural factors. Systematic study focusing on different biomass components will be required to assess their interaction which might not always be linear.

## Conclusions

The study established that characterization of bulk biomass composition based on wet chemical methods cannot explain differences in enzymatic biomass digestibility in response to differences in pretreatment severity and notably cannot provide unequivocal clues to explain differences in enzymatic digestibility (recalcitrance to enzymatic digestion) among different types of lignocellulosic biomass feedstocks—even among three types of stalk or stover biomass. Methods characterizing physical and chemical features of the biomass surface were more successful, namely contact angle measurement (wettability) and attenuated total reflectance-Fourier transform infrared (ATR-FTIR) spectroscopy (surface biopolymer composition). Higher surface content of hemicellulose, lignin, and wax promoted lower wettability (as seen by higher contact angle) therefore restraining the transport of water and enzyme and thus decreasing digestibility. Consequently, to a large extent, factors related to surface physical and/or chemical properties rather than bulk chemical composition seem to determine recalcitrance of biomass feedstocks of the types studied here. This conclusion emphasizes the fact that since the first contact of enzymes with biomass material is on the surface; studying the interaction between biomass and enzymes also requires understanding of multi-component interactions on the surface level. The molecular mechanisms and quantitative enzymatic conversion rate kinetics underlying these variations in surface properties need to be investigated further in order to understand lignocellulosic biomass recalcitrance better as well as to develop approaches to overcome it.

## Methods

### Biomass feedstocks

Wheat straw (*Triticum aestivum* L.) (WS) and *Miscanthus* × *giganteus* stalks (MS) were harvested at Aarhus University (AU) Foulum in autumn 2014. Corn stover (*Zea mays* subsp. *mays* L.) (CS) was harvested at AU Jyndevad in autumn 2014. CS and MS were stored frozen due to high moisture content. WS was dried on the field and stored dry at room temperature.

### Pretreatment conditions

HTP was performed using the Mini-IBUS equipment (Technical University of Denmark, Risø Campus). The biomass feedstocks were used as provided—no further milling or drying. The details of materials and pretreatment conditions are listed in Table 4. The severity factor log *R*_0_ (Eq. 1) is calculated according to Overend and Chornet [11]. Wheat straw was mixed with water and pre-conditioned in plastic bags over night at room temperature to obtain 40% dry matter (DM). Other biomass feedstocks were used directly due to high moisture content. After pretreatment, the biomass is pressed inside the reactor to obtain a solid fraction with around 35–40% DM and a liquid fraction. For each biomass and pretreatment condition, a minimum of three batches were done, each with a biomass loading of 1 kg DM. After pretreatment, the solid material from all three (or more) batches were mixed and then immediately frozen.

**Table 4:** HTP conditions used

Biomass feedstock	Pretreatment conditions		
	Temperature (°C)	Time (min)	Severity factor (log *R*_0_)
CS	190	10	3.65
	190	15	3.83
	195	15	3.97
MS	190	10	3.65
	190	15	3.83
	195	15	3.97
WS	190	10	3.65
	190	15	3.83
	195	15	3.97

CS, corn stover; MS, *Miscanthus* × *giganteus* stalks; WS, wheat straw

1 $$\log R_{0} = \log \left[ {t (\hbox{min} ) \times \left( {\frac{{T(^\circ {\text{C}}) - 100}}{14.75}} \right)} \right]$$

### Compositional analysis

Composition of biomass fiber fraction was determined using strong acid hydrolysis procedure [29]. Solvent extraction was performed on untreated biomass whereas the pretreated biomass feedstocks were washed with distilled water prior to strong acid hydrolysis.

The percentage removal of hemicellulose from the fiber fraction was determined by taking into account the chemical composition (i.e., content of arabinose and xylose calculated as dehydrated moieties) and the dry amount of biomass before and after pretreatment (Eq. 2):

2 $$\% \;{\text{removal}} = \frac{{{\text{hemicellulose}}\;{\text{in}}\;{\text{raw}}\;{\text{biomass }}\left( {{\text{g}}\,{\text{DM}}} \right) - {\text{hemicellulose}}\;{\text{in}}\;{\text{pretreated}}\;{\text{biomass}}\,({\text{g DM}})}}{{{\text{hemicellulose}}\;{\text{in}}\;{\text{raw}}\;{\text{biomass}} \,\left( {\text{g DM}} \right)}} \times 100$$

### Enzymatic hydrolysis

The pretreated biomass feedstocks were washed using distilled water prior to hydrolysis experiments and stored frozen until use. The washed pretreated biomass feedstocks were then homogenized in a blender BL-1200 (AS Wilfa, Skytta, Norway) for 3 min at medium setting with the corresponding buffer solution used in the hydrolysis experiment. The dry matter content of the slurry was adjusted to 1% DM based on measurement of DM (using a Moisture Content Analyzer HR83, Mettler Toledo GmbH, Greifensee, Switzerland). The slurry was always prepared fresh for every experiment.

Enzymatic hydrolysis of pretreated biomass feedstocks was performed in triplicates at 1% DM in 0.05 M acetate buffer pH 5.0 at 50 °C with Cellic® CTec3 (Novozymes A/S, Bagsværd, Denmark). The hydrolysis experiment was carried out in 2 ml Protein LoBind® tubes (Eppendorf AG, Hamburg, Germany) and agitated with vortex mixing at 1250 RPM using ThermoMixer Comfort (Eppendorf AG, Hamburg, Germany). Dosage response curve experiments were performed at a constant duration of 72 h with enzyme dosage of 5, 10, 20, and 60 mg protein/g DM biomass. Reactions were halted by boiling samples for 10 min. After centrifugation, reaction mixture supernatants were analyzed for monosaccharides and corrected with biomass and enzyme blanks as reference.

### Analysis of monosaccharides

Monosaccharides were quantified by high performance anion exchange chromatography with pulsed amperometric detection (HPAEC-PAD) using a Dionex ICS-5000 system (DionexCorp, Sunnyvale, CA, USA) equipped with a CarboPac PA1 analytical column (250 × 4 mm) and a CarboPac PA1 guard column (250 × 4 mm) operated at a flow rate of 1 ml/min. Isocratic elution took place at 25 °C, with water, for 30 min. The column was then washed for 10 min with 500 mM NaOH and equilibrated with water for 10 min. Detection was done by post-column addition of 500 mM NaOH at 0.2 ml/min. Standards of d-glucose, d-xylose, l-arabinose, d-galactose, and d-mannose were used for quantification.

## 2D Nuclear magnetic resonance (NMR)

Untreated and fiber fraction of pretreated biomass feedstocks was prepared in DMSO-d_6_/pyridine-d_5_ (4:1, v/v) according to a protocol for whole plant cell wall characterization [40]. Heteronuclear single quantum coherence (HSQC) experiments of samples were performed using a 600 MHz Avance III HD (Bruker, Billerica, MA, USA) equipped with a cryogenically cooled 5 mm dual probe optimized for ^13^C and ^1^H. For ^13^C-^1^H HSQC NMR, 16 scans of the Bruker pulse sequence hsqcetpg was applied with a fixed spectral width of 220 ppm for ^13^C and 13 ppm for ^1^H. The NMR spectra were analyzed and processed using Bruker’s Topspin 3.5 software. The central DMSO solvent peaks were used as internal reference (*δ*_H_/*δ*_C_ = 2.50/39.50). Peak assignment was done according to previously reported peaks [40] and contour volume integration was done at lowest contour level method.

### Comprehensive microarray polymer profiling (CoMPP)

The procedure is performed based on previously established works [44, 48, 70]. Alcohol insoluble residue (AIR) was prepared from untreated and solid fraction of pretreated biomass feedstocks by freeze-drying and ball-milling. Milled tissue was washed five times in pre-warmed 70% v/v ethanol, shaking for 10 min and pelleted by centrifugation, followed by washing with acetone. For the CoMPP analysis, the liquid fractions of pretreated biomass feedstocks were used directly, while the solid fractions were sequentially extracted.

The extraction was performed first with 50 mM diaminocyclohexanetetraacetic acid (CDTA) for predominant pectin extraction, then with alkaline 4 M NaOH in 0.1% (w/v) NaBH_4_ that extracts mainly hemicelluloses. Both liquid and extracted samples were spotted using a microarray robot (Sprint, Arrayjet, Roslin, UK). Once printed, arrays were blocked with phosphate-buffered saline (PBS) containing 5% (w/v) low fat milk powder (MPBS). Arrays were washed with PBS and probed with antibodies (PlantProbes, Leeds University, UK) in 5% MPBS. The antibodies used were BS-400-3 that targets mixed linked glucan (MLG); LM10, LM11, and LM23 that target xylans; LM15 and LM25 that target xyloglucans; LM21 and BS-400-4 that target mannans [45, 46].

Subsequently, the arrays were washed in PBS and incubated with anti-rat secondary antibody conjugated to alkaline phosphatase (Sigma, St. Louis, USA) in 5% (w/v) MPBS 1/5000. Arrays were developed in a solution containing 5-bromo-4-chloro-3-indolylphosphate and nitro blue tetrazolium in alkaline phosphatase buffer (100 mM NaCl, 5 mM MgCl_2_, 100 mM diethanolamine, pH 9.5). Developed microarrays were scanned at 2400 dpi (CanoScan 8800 F, Canon, Søborg, Denmark) and converted to TIFFs. Antibody signals were measured using appropriate software (Array-Pro Analyzer 6.3, Media Cybernetics, Rockville, USA). Data were presented as two datasets where maximal spot signal was set to 100 and all other values normalized accordingly with color intensity is correlating with mean spot signal value.

### Wettability test

Wettability of biomass feedstocks was assessed by measuring initial water contact angle according to Heiss-Blanquet et al. [28]. The untreated and pretreated biomass feedstocks were air-dried at room temperature and milled using a MF 10 microfine grinder (IKA® Werke GmbH & Co. KG, Staufen, Germany) to obtain particles that pass a 0.5 mm sieve. The milled biomass samples (2 g DM) were then pressed at tonnage load of 10 Mg for 5 min using an Atlas Manual 25 T Hydraulic Press (Specac ltd., Kent, UK), yielding a tablet with diameter of 4 cm. Ultrapure water was used for the sessile drop method at controlled working temperature of 22.0 °C using OCA 20 instrument (DataPhysics Instruments GmbH, Filderstadt, Germany). The water drop (15 μl) was deposited to the surface of the pellet using a computer-controlled syringe and the images were recorded at 2.5 frames per second. The images of drop shape were analyzed using SCA 20 software (DataPhysics Instruments GmbH, Filderstadt, Germany) to calculate the initial contact angle using Young–Laplace fitting mode. The wettability test was performed with five replicates for each tablet.

### Attenuated total reflectance-Fourier transform infrared (ATR-FTIR) spectroscopy

Air-dried untreated and pretreated biomass feedstocks were milled using a MF 10 microfine grinder (IKA® Werke GmbH & Co. KG, Staufen, Germany) to obtain particles that passed 0.5 mm sieve. ATR-FTIR measurements were performed with five replicates using a Nicolet 6700 FT-IR, Pike Technologies GladiATR diamond spectrometer (Thermo Scientific, Waltham, MA, USA), with a working temperature of 25 °C. The spectral range included was 4000–600 cm^−1^ and spectra were obtained using 64 scans (128 for the background) and a resolution of 4.0 cm^−1^. Peak areas were estimated based on the trapz algorithm as implemented in Matlab R2014A (The Mathworks Inc., Natick, MA, USA). Individual linear baselines were applied for each peak. The peaks included are listed in Table 5. In order to provide semi-quantitative analysis of the surface chemical composition, ratios of peak areas were calculated for 1508/895 cm^−1^ corresponding to lignin/holocellulose, 1732/895 cm^−1^ corresponding to hemicellulose/holocellulose and 2918/895 cm^−1^ corresponding to wax/holocellulose. Therefore, the peak area ratios represent apparent surface abundance (ASA) of the corresponding components, i.e., apparent surface abundance of lignin relative to holocellulose (ASA-L/C), hemicellulose relative to holocellulose (ASA-H/C) and wax relative to holocellulose (ASA-W/C).

**Table 5:** ATR-FTIR assignments of wavenumbers used to measure peak area

Wavenumber (cm^−1^)	Asssignment^a^		Estimated penetration depth^a^ (μm)
895	Holocellulose	Anomeric C-groups, C_1_-H deformation, ring valence vibration (cellulose, wood, holocellulose) [71]	1.85
1508	Lignin	Aromatic skeletal vibrations [71, 72]	1.10
1732	Hemicellulose	C=O stretch in unconjugated carbonyl groups of carbohydrate origin (side chain acetylation in mannan, carboxylic acid side chain in xylan, and ester groups in lignin-carbohydrate complexes) [71, 72]	0.96
2918	Wax	Asymmetric CH_2_ stretching from cuticular waxes [73]	0.57

^a^Calculated based on the formula (Eq. 3):

$$d_{\text{p}} = \frac{\lambda }{{2\pi n_{1} \sqrt {\sin^{2} \theta - \left( {n_{2} /n_{1} } \right)^{2} } }},$$        (3)

where *d*_p_, *λ*, *θ*, *n*_1_, and *n*_2_ are penetration depth, wavelength, incident angle, ATR crystal refractive index, and sample refractive index, respectively. The values of *θ* and *n*_1_ are known specifically to be 45° and 2.40, respectively, for diamond ATR. The refractive index of biomass samples is estimated to be 1.4 which is a common value for organic polymer, e.g., in wood cell wall [74]

### Statistical analysis

One-way analysis of variance (ANOVA) was performed using JMP 12 (SAS Institute Inc., Cary, NC, USA) with post hoc analysis using Tukey–Kramer’s Honestly Significant Difference (HSD) test at *p* ≤ 0.05. Least-squares linear regression analyses of the scatter plots were performed using OriginPro 2016 (OriginLab Corp., Northampton, MA, USA) using York linear fitting to account for errors in both *x*- and *y*-axes. The trend and significance of the relationship between the data were validated using Pearson’s correlation coefficient (*R*) and *t* test for the slope value where significant relationship is indicated by *p* value <0.05.

## Additional file

**Additional file 1: Figures S1–S12.** ^13^C-^1^H HSQC (heteronuclear single quantum coherence) spectra of untreated (raw) and hydrothermally pretreated (log *R*_0_ = 3.65, 3.83 and 3.97) corn stover, *Miscanthus* × *giganteus* stalks and wheat straw. **Figure S13.** Phenylcoumaran structure. **Figures S14–S16.** Selected ATR-FTIR spectra each representing sample from untreated (raw) and hydrothermally pretreated (log *R*_0_ = 3.65, 3.83 and 3.97) corn stover, *Miscanthus* × *giganteus* stalks and wheat straw. **Figures S17–S30.** Scatter plot of surface and bulk chemical composition with glucose release and wettability test of hydrothermally pretreated (log *R*_0_ = 3.65, 3.83 and 3.97) corn stover, *Miscanthus* × *giganteus* stalks and wheat straw.

## Abbreviations

ANOVA: one-way analysis of variance
ASA-H/C: apparent surface abundance of hemicellulose relative to holocellulose
ASA-L/C: apparent surface abundance of lignin relative to holocellulose
ASA-W/C: apparent surface abundance of wax relative to holocellulose
ATR-FTIR: attenuated total reflectance-Fourier transform infrared (spectroscopy)
AX: arabinoxylan
^13^C-^1^H HSQC: heteronuclear single quantum coherence (spectra)
CAM: contact angle measurement
CoMPP: comprehensive microarray polymer profiling
CS: corn stover
DM: dry matter
HTP: hydrothermal pretreatment
MLG: mixed linked glucan
MS: *Miscanthus* × *giganteus* stalks
NMR: nuclear magnetic resonance
WS: wheat straw
XG: xyloglucan
