# Supplementary material for: Surface properties correlate to the digestibility of hydrothermally pretreated lignocellulosic Poaceae biomass feedstocks
Source: Biotechnol Biofuels. 2017 Feb 23;10:49. doi: 10.1186/s13068-017-0730-3 (PMC5322652; doi:10.1186/s13068-017-0730-3)
Supplement: Supplementary file 1 — Additional file 1: Figures S1–S12. 13C-1H HSQC (heteronuclear single quantum coherence) spectra of untreated (raw) and hydrothermally pretreated (log R 0 = 3.65, 3.83 and 3.97) corn stover, Miscanthus × giganteus stalks and wheat straw. Figure S13. Phenylcoumaran structure. Figures S14–S16. Selected ATR-FTIR spectra each representing sample from untreated (raw) and hydrothermally pretreated (log R 0 = 3.65, 3.83 and 3.97) corn stover, Miscanthus × giganteus stalks and wheat straw. Figures S17–S30. Scatter plot of surface and bulk chemical composition with glucose release and wettability test of hydrothermally pretreated (log R 0 = 3.65, 3.83 and 3.97) corn stover, Miscanthus × giganteus stalks and wheat straw. [file 13068_2017_730_MOESM1_ESM.docx]

Supplementary information for:

Surface properties correlate to the digestibility of hydrothermally pretreated lignocellulosic Poaceae biomass feedstocks

Demi T. Djajadi^1^, Aleksander R. Hansen^2^, Anders Jensen^3^, Lisbeth G. Thygesen^3^, Manuel Pinelo^1^, Anne S. Meyer^1*^, Henning Jørgensen^1**^

Affiliations:

1: Department of Chemical and Biochemical Engineering, Søltofts Plads Building 229, Technical University of Denmark, 2800 Kongens Lyngby, Denmark

2: Department of Plant and Environmental Sciences, Thorvaldsensvej 40, University of Copenhagen, 1871 Frederiksberg C, Denmark

3: Department of Geosciences and Natural Resource Management, Rolighedsvej 23, University of Copenhagen, 1958 Frederiksberg C, Denmark

*Corresponding author: Anne S. Meyer ([am@kt.dtu.dk](mailto:am@kt.dtu.dk))

**Present address: Department of Plant and Environmental Sciences, Thorvaldsensvej 40, University of Copenhagen, 1871 Frederiksberg C, Denmark


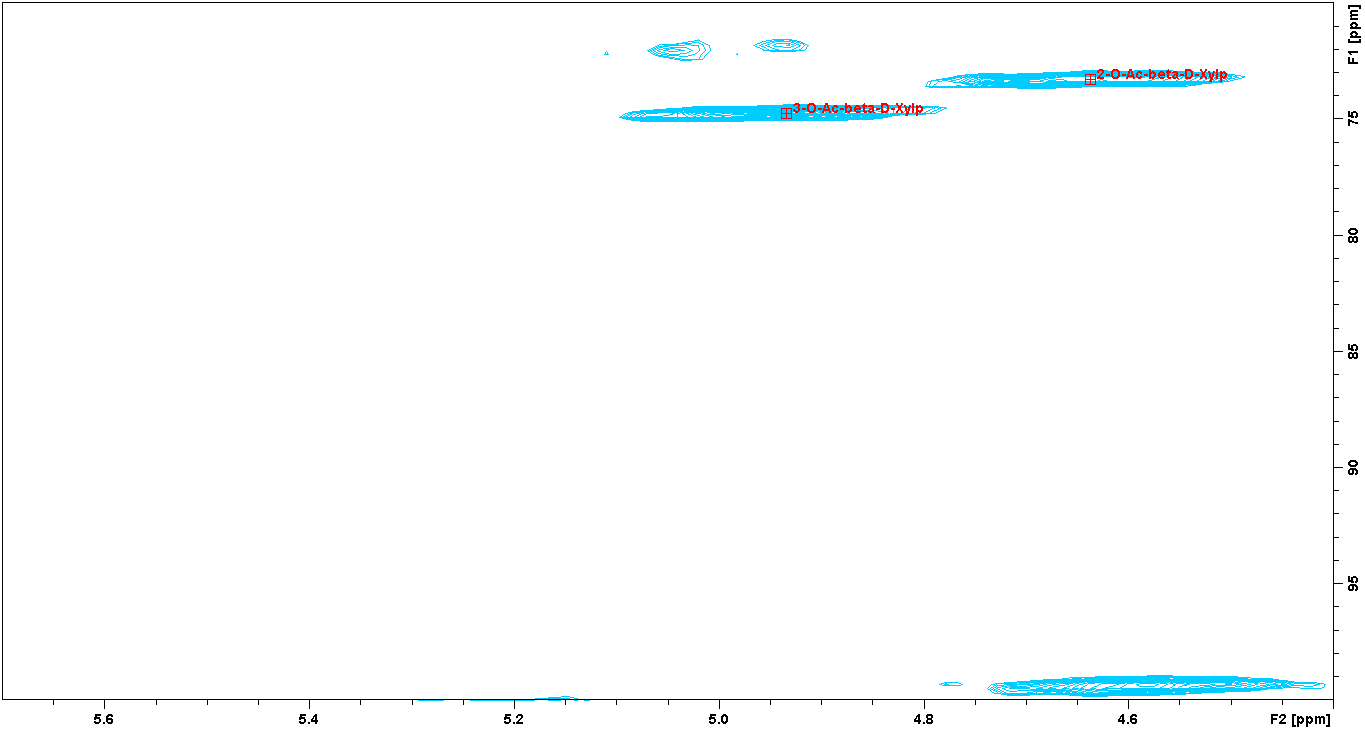


## Figure S1. ^13^C-^1^H HSQC (heteronuclear single quantum coherence) spectra of raw (untreated) corn stover displaying the peaks for acetylated positions (2-O-Ac-β-D-Xyl*p* and 3-O-Ac-β-D-Xyl*p*).


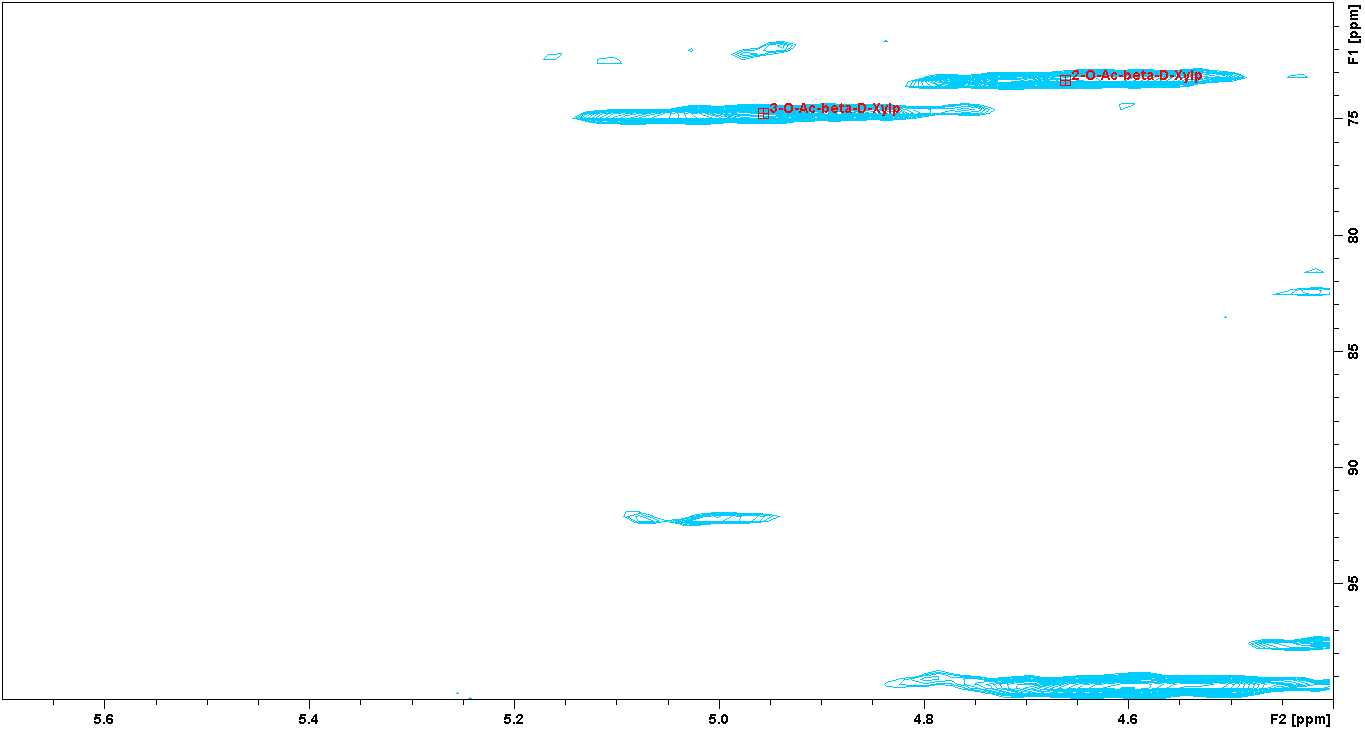


## Figure S2. 13C-1H HSQC (heteronuclear single quantum coherence) spectra of corn stover hydrothermally pretreated at log *R_0_* = 3.65 displaying the peaks for acetylated positions (2-O-Ac-β-D-Xylp and 3-O-Ac-β-D-Xylp).


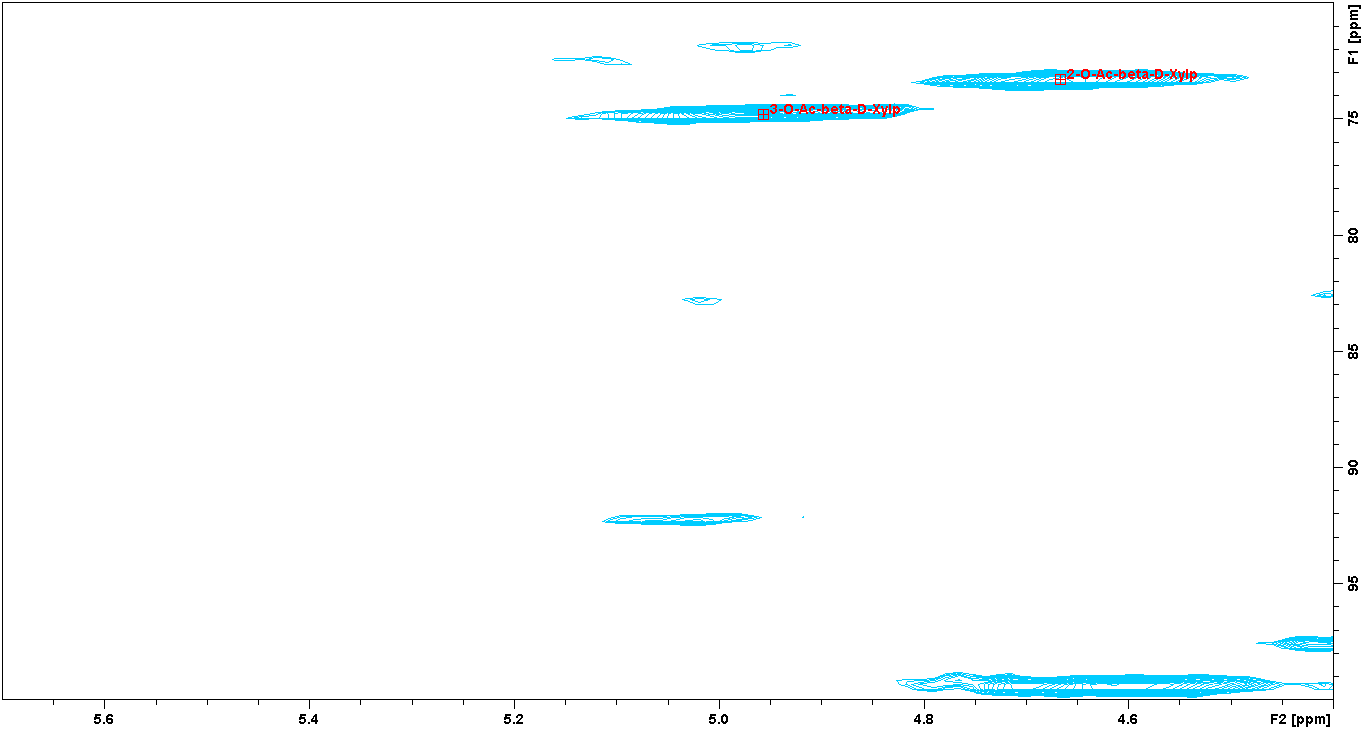


## Figure S3. ^13^C-^1^H HSQC (heteronuclear single quantum coherence) spectra of corn stover hydrothermally pretreated at log *R_0_* = 3.83 displaying the peaks for acetylated positions (2-O-Ac-β-D-Xyl*p* and 3-O-Ac-β-D-Xyl*p*).


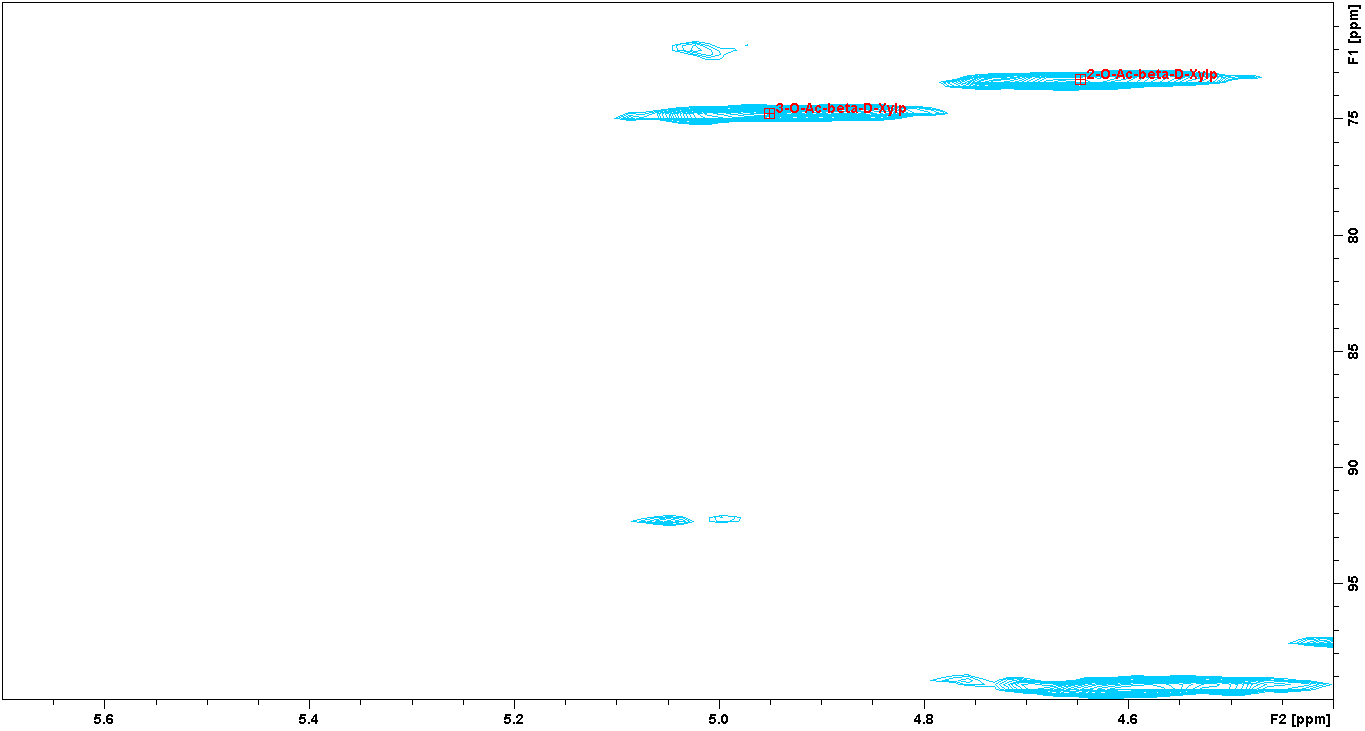


## Figure S4. ^13^C-^1^H HSQC (heteronuclear single quantum coherence) spectra of corn stover hydrothermally pretreated at log *R_0_* = 3.97 displaying the peaks for acetylated positions (2-O-Ac-β-D-Xyl*p* and 3-O-Ac-β-D-Xyl*p*).


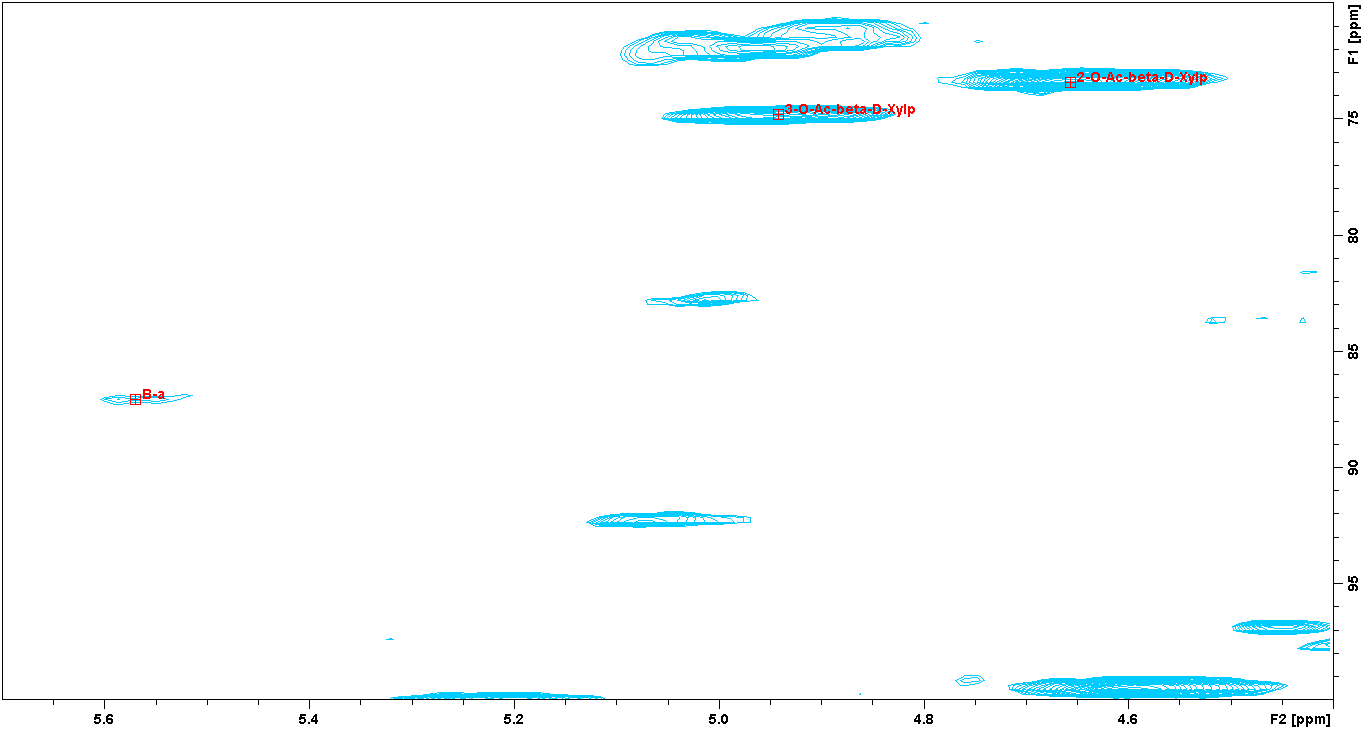


## Figure S5. ^13^C-^1^H HSQC (heteronuclear single quantum coherence) spectra of raw (untreated) *Miscanthus* × *giganteus* stalks displaying the peaks for acetylated positions (2-O-Ac-β-D-Xyl*p* and 3-O-Ac-β-D-Xyl*p*) relative to the phenylcoumaran-α (B-a).


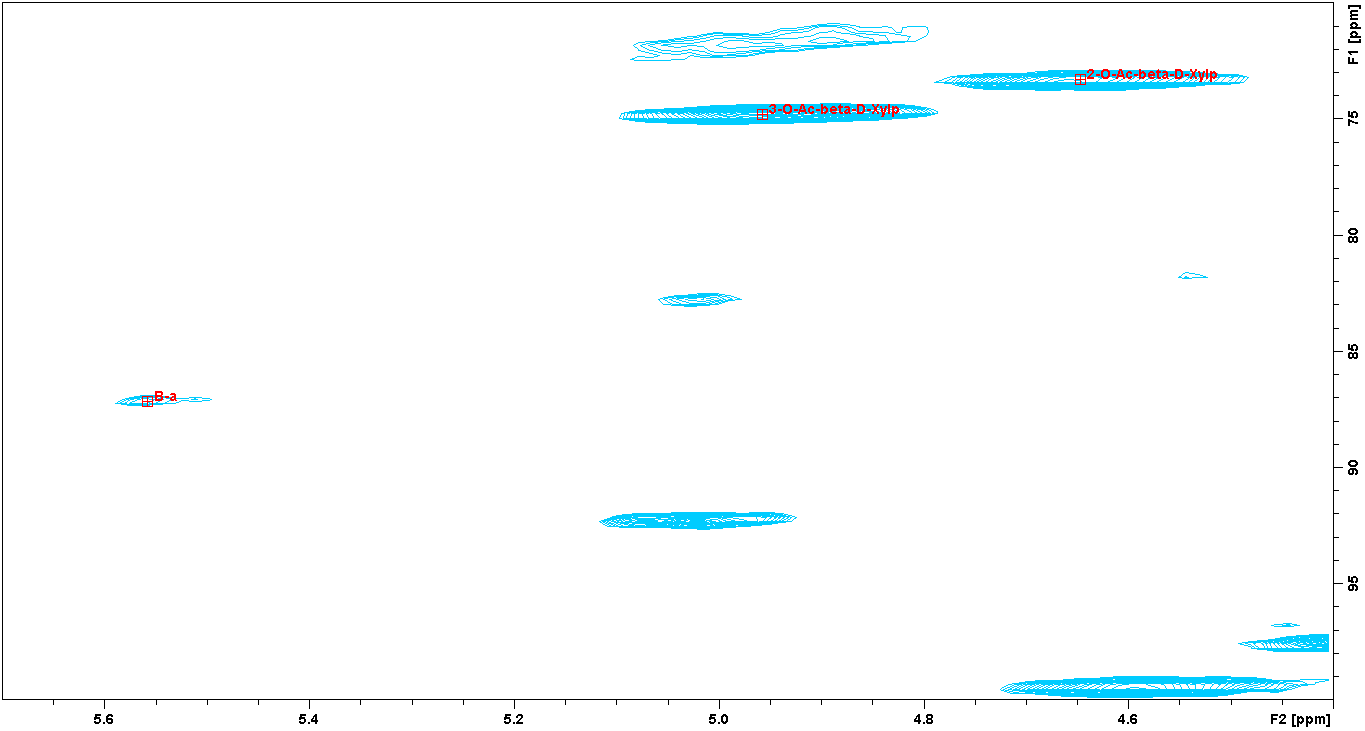


## Figure S6. ^13^C-^1^H HSQC (heteronuclear single quantum coherence) spectra of *Miscanthus* × *giganteus* stalks hydrothermally pretreated at log *R_0_* = 3.65 displaying the peaks for acetylated positions (2-O-Ac-β-D-Xyl*p* and 3-O-Ac-β-D-Xyl*p*) relative to the phenylcoumaran-α (B-a).


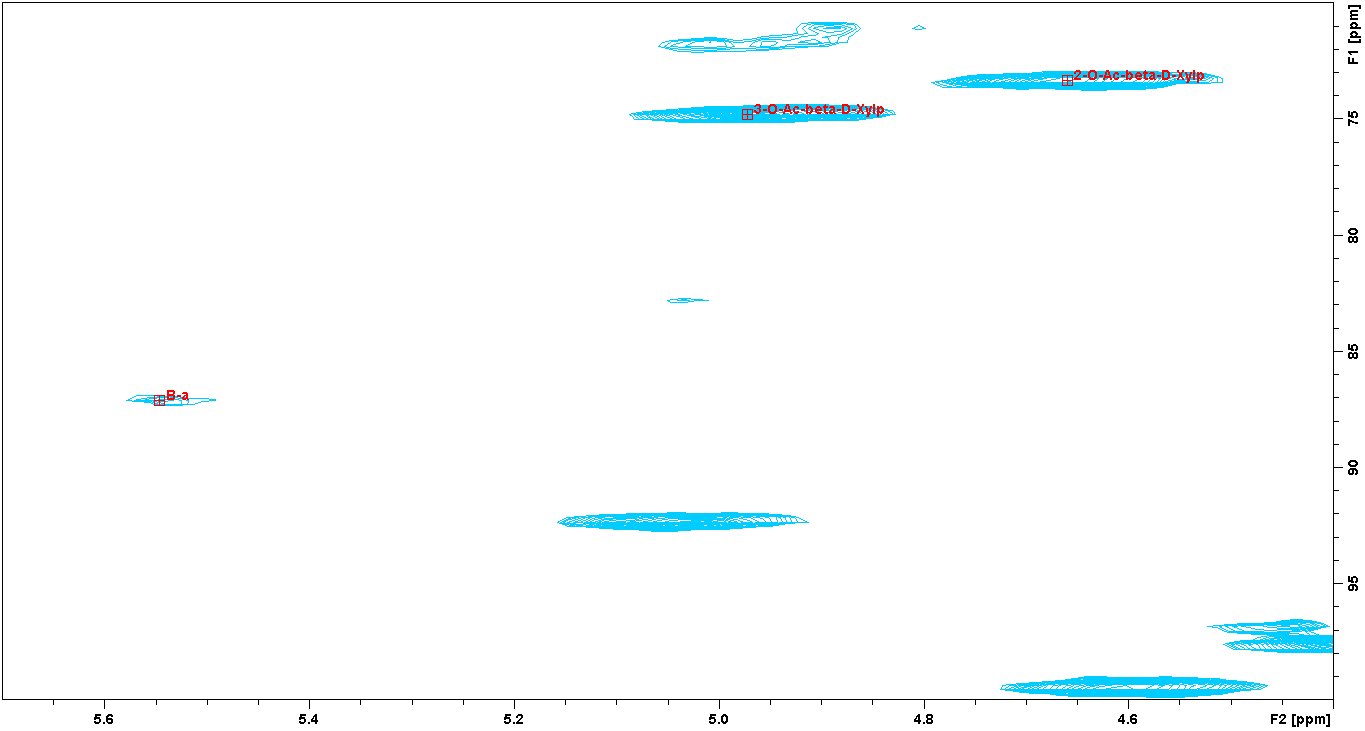


## Figure S7. ^13^C-^1^H HSQC (heteronuclear single quantum coherence) spectra of *Miscanthus* × *giganteus* stalks hydrothermally pretreated at log *R_0_* = 3.83 displaying the peaks for acetylated positions (2-O-Ac-β-D-Xyl*p* and 3-O-Ac-β-D-Xyl*p*) relative to the phenylcoumaran-α (B-a).


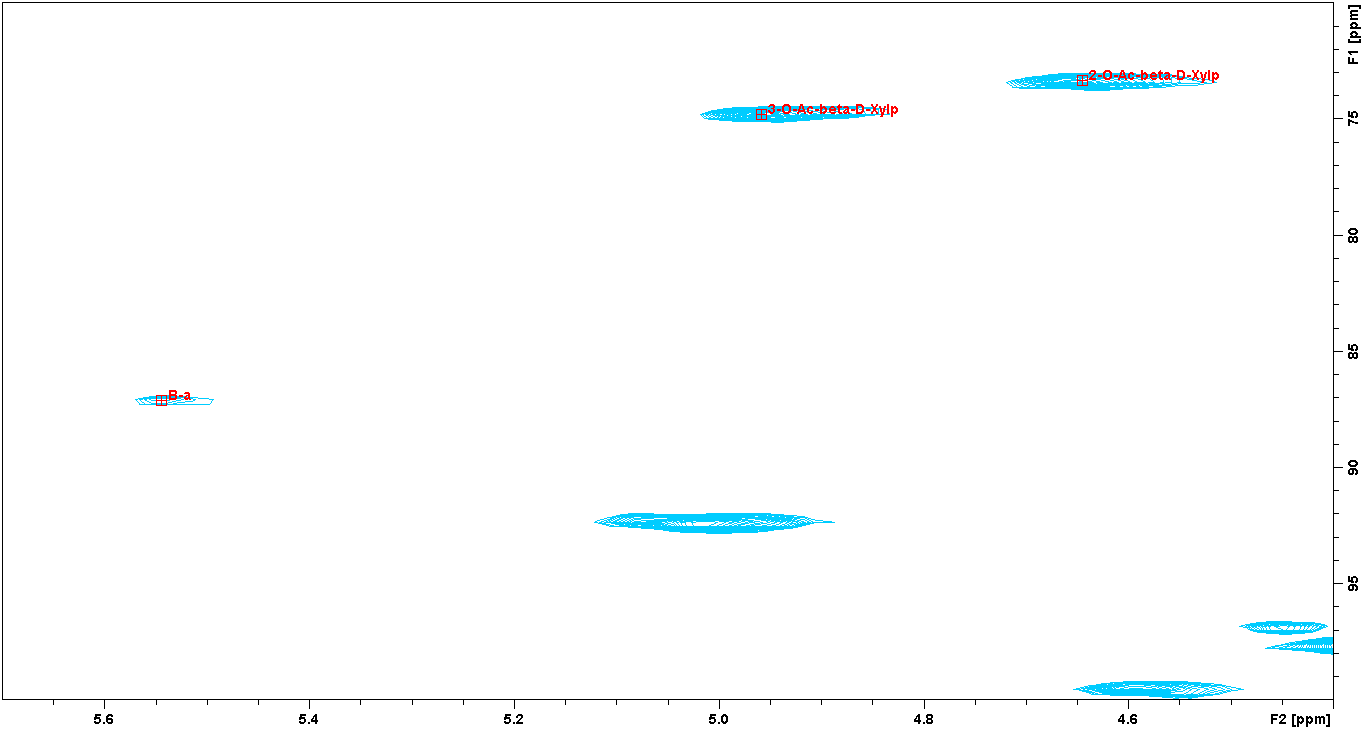


## Figure S8. ^13^C-^1^H HSQC (heteronuclear single quantum coherence) spectra of *Miscanthus* × *giganteus* stalks hydrothermally pretreated at log *R_0_* = 3.97 displaying the peaks for acetylated positions (2-O-Ac-β-D-Xyl*p* and 3-O-Ac-β-D-Xyl*p*) relative to the phenylcoumaran-α (B-a).


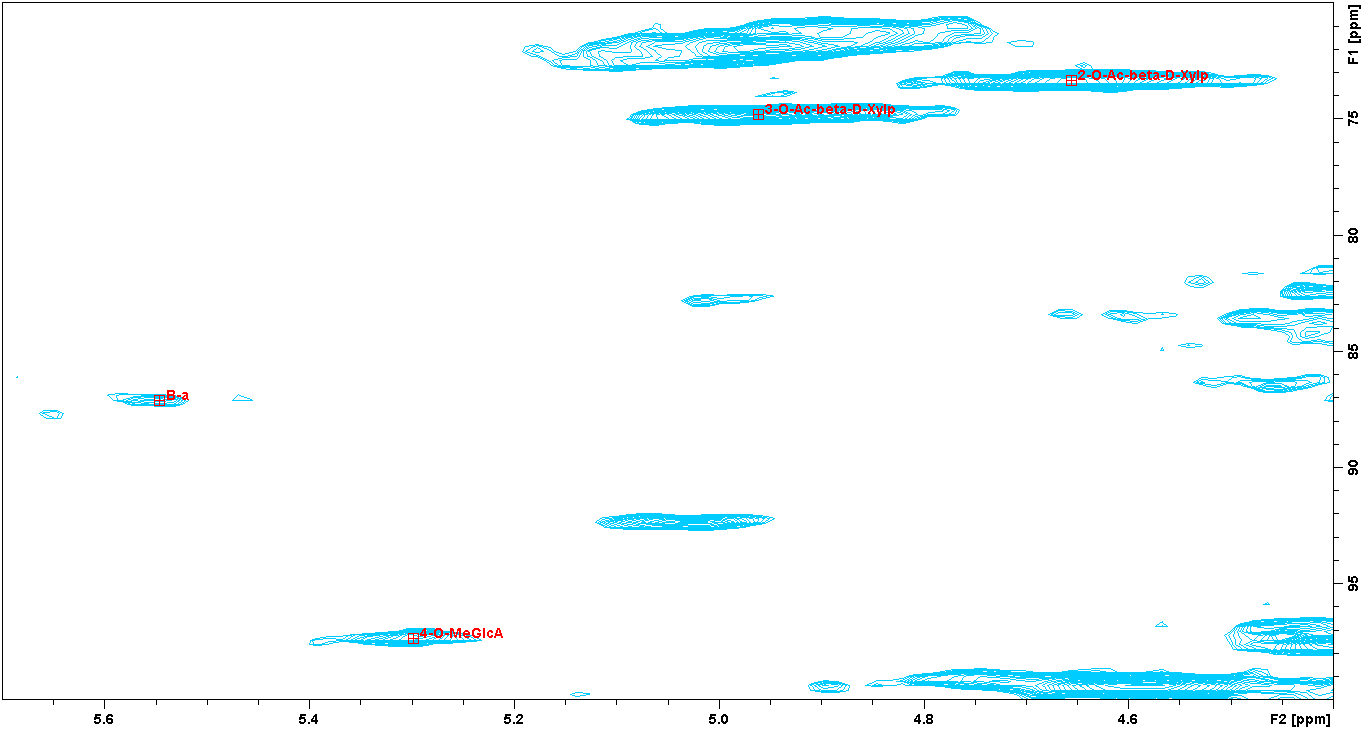


## Figure S9. ^13^C-^1^H HSQC (heteronuclear single quantum coherence) spectra of raw (untreated) wheat straw displaying the peaks for acetylated positions (2-O-Ac-β-D-Xyl*p* and 3-O-Ac-β-D-Xyl*p*), 4-*O*-methyl glucuronosyl residues (4-O-MeGlcA) relative to the phenylcoumaran-α (B-a).


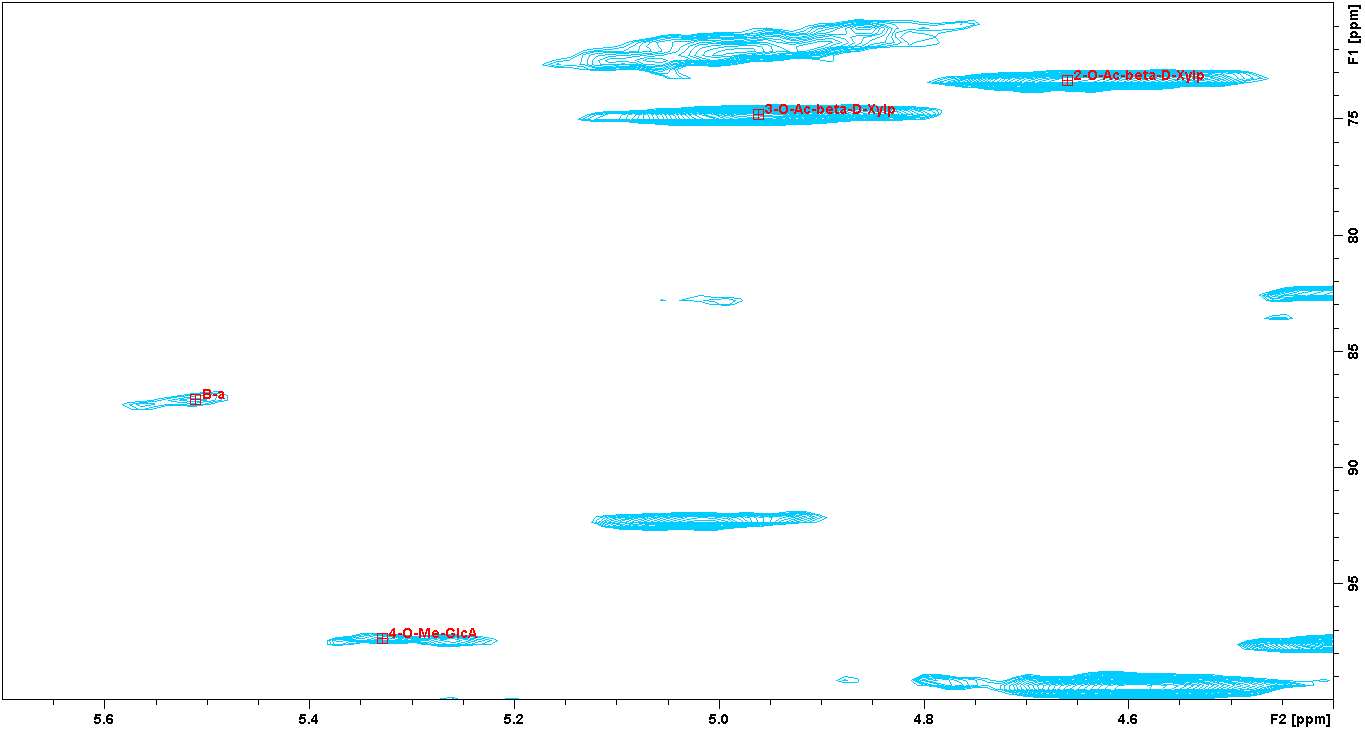


## Figure S10. ^13^C-^1^H HSQC (heteronuclear single quantum coherence) spectra of wheat straw hydrothermally pretreated at log *R_0_* = 3.65 displaying the peaks for acetylated positions (2-O-Ac-β-D-Xyl*p* and 3-O-Ac-β-D-Xyl*p*), 4-*O*-methyl glucuronosyl residues (4-O-MeGlcA) relative to the phenylcoumaran-α (B-a).


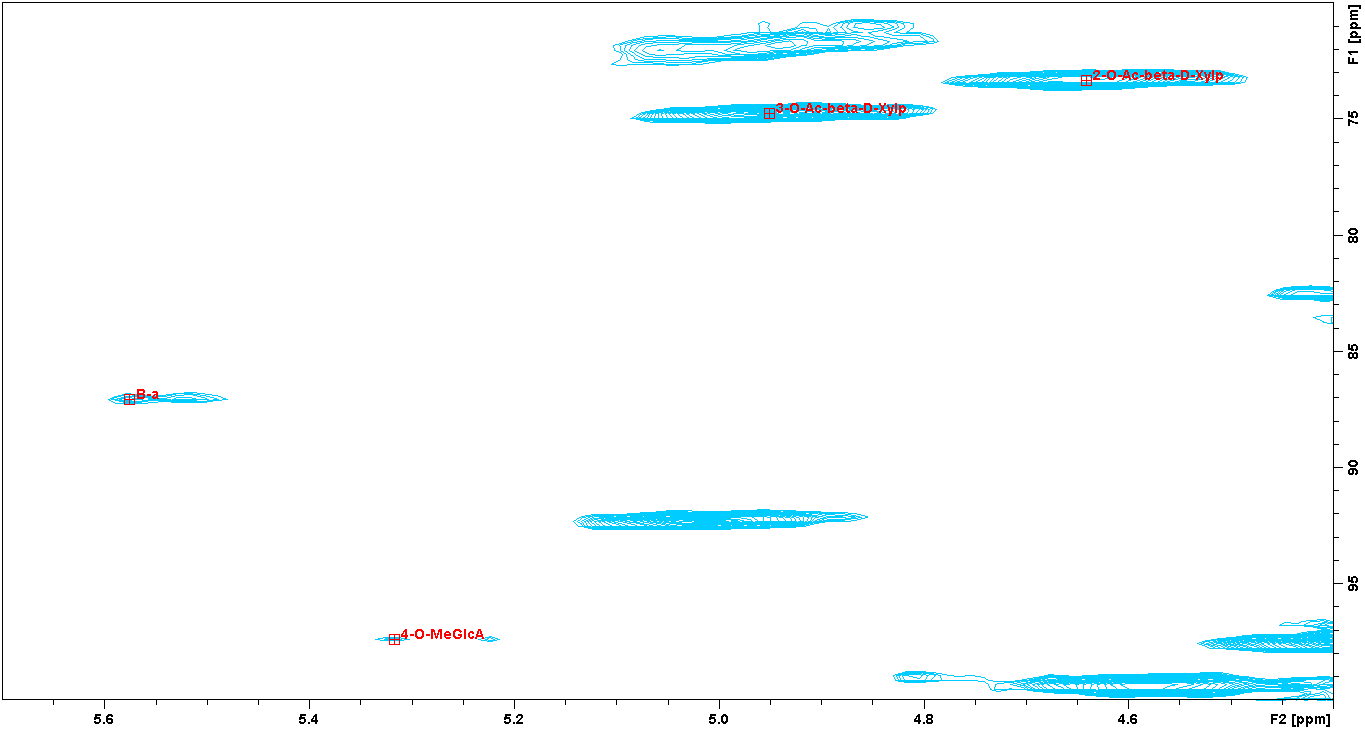


## Figure S11. ^13^C-^1^H HSQC (heteronuclear single quantum coherence) spectra of wheat straw hydrothermally pretreated at log *R_0_* = 3.83 displaying the peaks for acetylated positions (2-O-Ac-β-D-Xyl*p* and 3-O-Ac-β-D-Xyl*p*), 4-*O*-methyl glucuronosyl residues (4-O-MeGlcA) relative to the phenylcoumaran-α (B-a).


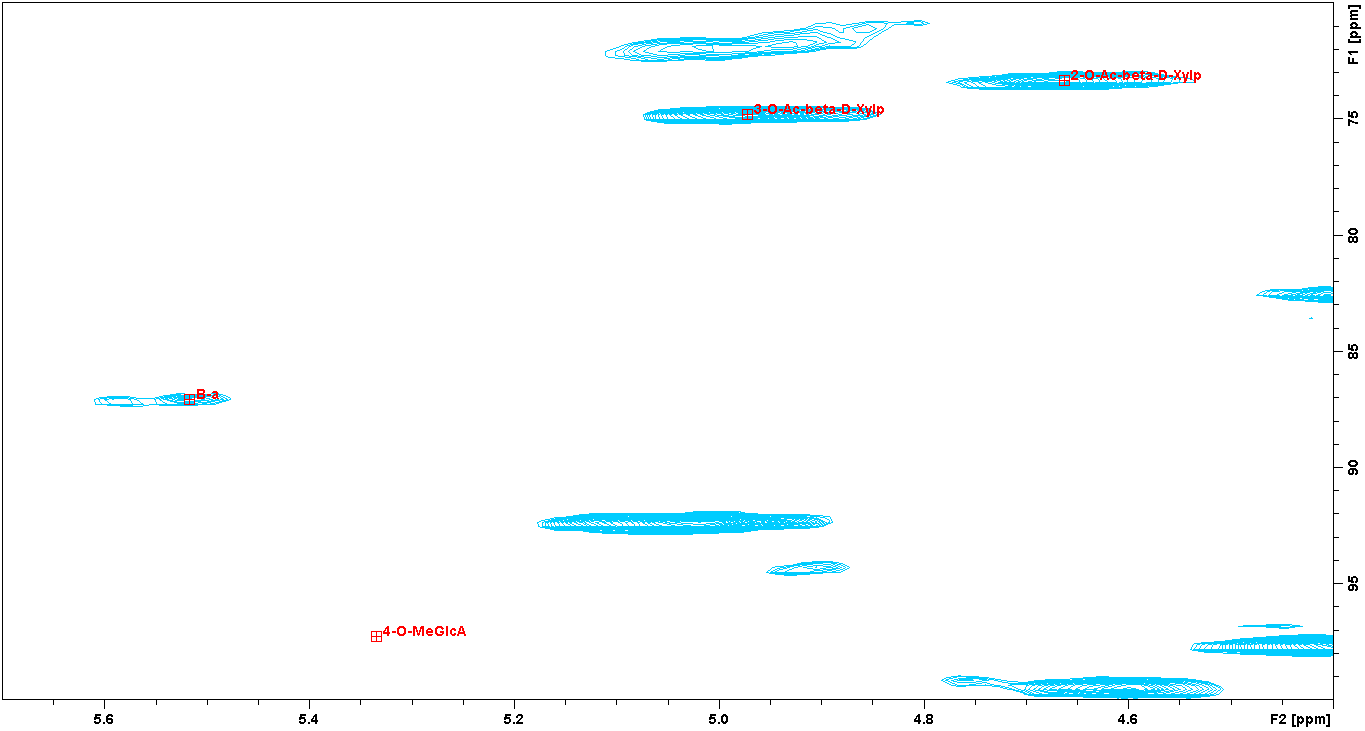


## Figure S12. ^13^C-^1^H HSQC (heteronuclear single quantum coherence) spectra of wheat straw hydrothermally pretreated at log *R_0_* = 3.97 displaying the peaks for acetylated positions (2-O-Ac-β-D-Xyl*p* and 3-O-Ac-β-D-Xyl*p*), 4-*O*-methyl glucuronosyl residues (4-O-MeGlcA) relative to the phenylcoumaran-α (B-a).


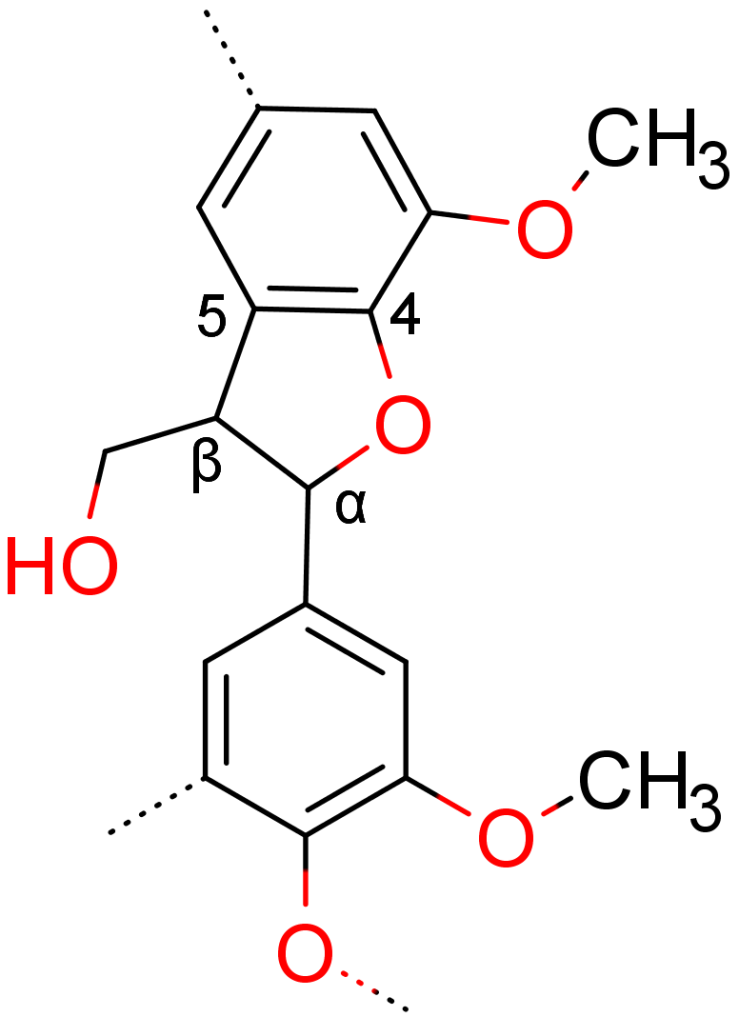


## Figure S13. Phenylcoumaran [β-5 (α-O-4)] structure; one of the major sub-structural units in the lignin polymer [1,2]. C_α_-H_α_ peak signal (phenylcoumaran-α) was used as reference during contour volume integration. The chemical structure was drawn using MarvinSketch 16.4.4.0 (ChemAxon Ltd., Budapest, Hungary).


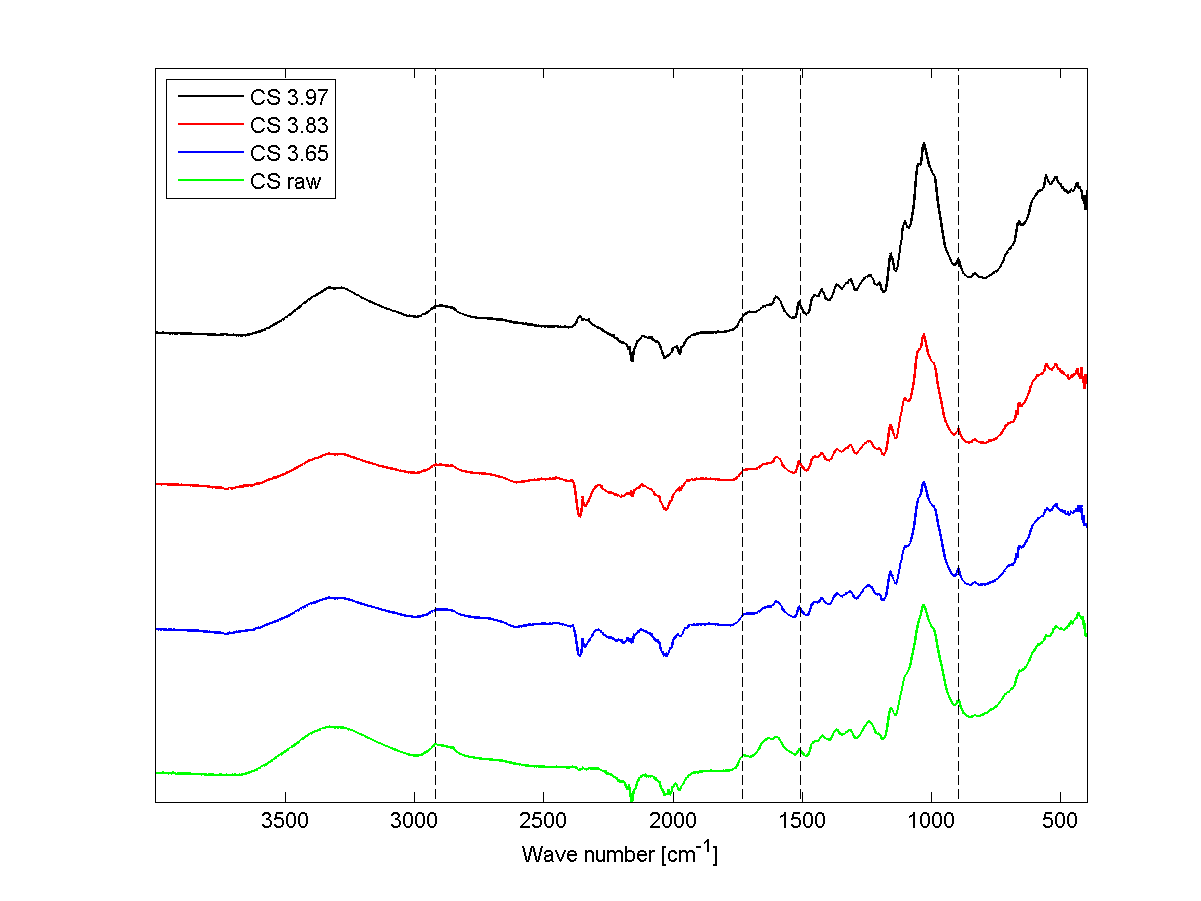


## Figure S14. Selected ATR-FTIR spectra each representing sample from raw (untreated) and hydrothermally pretreated (log *R_0_* = 3.65, 3.83 and 3.97) corn stover (CS). The vertical dashed lines mark the positions of the bands at 2918 (ascribed to wax), 1732 (ascribed to hemicellulose), 1508 (ascribed to lignin) and 895 cm^-1^ (ascribed to holocellulose).


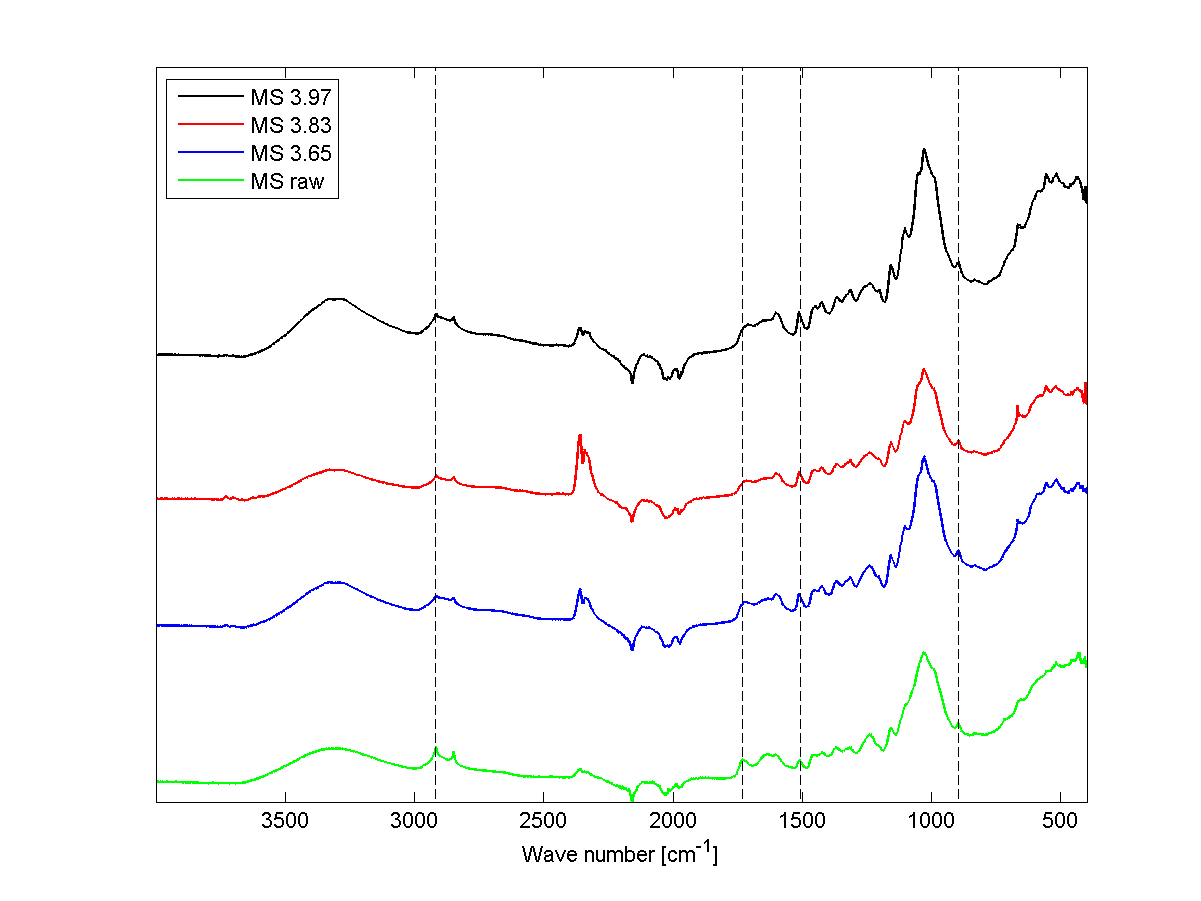


## Figure S15. Selected ATR-FTIR spectra each representing sample from raw (untreated) and hydrothermally pretreated (log *R_0_* = 3.65, 3.83 and 3.97) *Miscanthus* × *giganteus* stalks (MS). The vertical dashed lines mark the positions of the bands at 2918 (ascribed to wax), 1732 (ascribed to hemicellulose), 1508 (ascribed to lignin) and 895 cm^-1^ (ascribed to holocellulose).


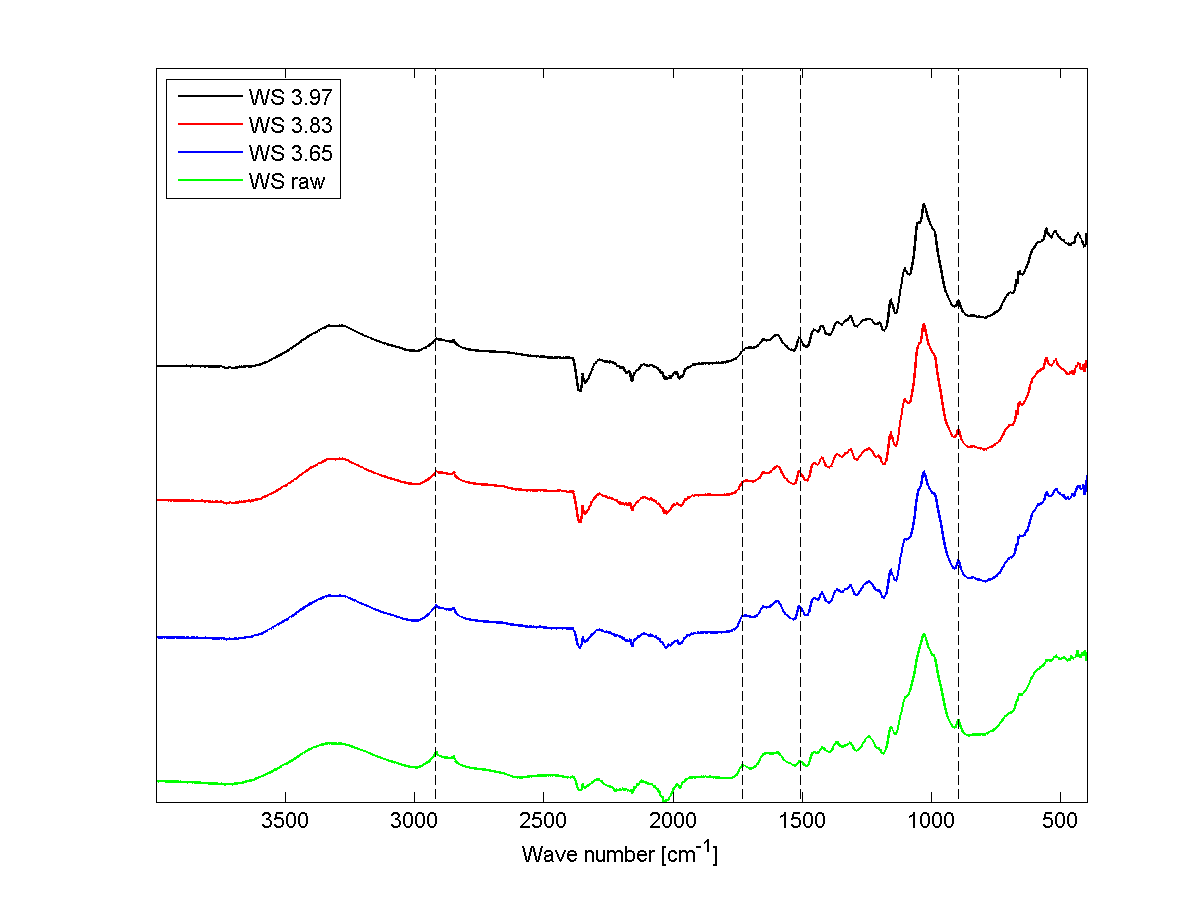


## Figure S16. Selected ATR-FTIR spectra each representing sample from raw (untreated) and hydrothermally pretreated (log *R_0_* = 3.65, 3.83 and 3.97) wheat straw (WS). The vertical dashed lines mark the positions of the bands at 2918 (ascribed to wax), 1732 (ascribed to hemicellulose), 1508 (ascribed to lignin) and 895 cm^-1^ (ascribed to holocellulose).





## Figure S17. Scatter plot of the hemicellulose (1732 cm^-1^) peak area ratio relative to that of holocellulose (895 cm^-1^) from ATR-FTIR and the glucose release after 72 h enzymatic hydrolysis of corn stover, *Miscanthus* × *giganteus* stalks and wheat straw hydrothermally pretreated at log *R_0_* = 3.65, 3.83 and 3.97 with 10 mg/g enzyme dosage.





## Figure S18. Scatter plot of the lignin (1508 cm^-1^) peak area ratio relative to that of holocellulose (895 cm^-1^) from ATR-FTIR and the glucose release after 72 h enzymatic hydrolysis of corn stover, *Miscanthus* × *giganteus* stalks and wheat straw hydrothermally pretreated at log *R_0_* = 3.65, 3.83 and 3.97 with 10 mg/g enzyme dosage.





## Figure S19. Scatter plot of the wax (2918 cm^-1^) peak area ratio relative to that of holocellulose (895 cm^-1^) from ATR-FTIR and the glucose release after 72 h enzymatic hydrolysis of corn stover, *Miscanthus* × *giganteus* stalks and wheat straw hydrothermally pretreated at log *R_0_* = 3.65, 3.83 and 3.97 with 10 mg/g enzyme dosage.





## Figure S20. Scatter plot of cellulose (glucan) content from composition analysis and the glucose release after 72 h enzymatic hydrolysis of corn stover, *Miscanthus* × *giganteus* stalks and wheat straw hydrothermally pretreated at log *R_0_* = 3.65, 3.83 and 3.97 with 10 mg/g enzyme dosage.





## Figure S21. Scatter plot of hemicellulose (arabino-/xylan) content from composition analysis and the glucose release after 72 h enzymatic hydrolysis of corn stover, *Miscanthus* × *giganteus* stalks and wheat straw hydrothermally pretreated at log *R_0_* = 3.65, 3.83 and 3.97 with 10 mg/g enzyme dosage.





## Figure S22. Scatter plot of lignin content from composition analysis and the glucose release after 72 h enzymatic hydrolysis of corn stover, *Miscanthus* × *giganteus* stalks and wheat straw hydrothermally pretreated at log *R_0_* = 3.65, 3.83 and 3.97 with 10 mg/g enzyme dosage.





## Figure S23. Scatter plot of hemicellulose (arabinose and xylose) removal from composition analysis and the glucose release after 72 h enzymatic hydrolysis of corn stover, *Miscanthus* × *giganteus* stalks and wheat straw hydrothermally pretreated at log *R_0_* = 3.65, 3.83 and 3.97 with 10 mg/g enzyme dosage.





## Figure S24. Scatter plot of the hemicellulose (1732 cm^-1^) peak area ratio relative to that of holocellulose (895 cm^-1^) from ATR-FTIR and the initial water contact angle of corn stover, *Miscanthus* × *giganteus* stalks and wheat straw hydrothermally pretreated at log *R_0_* = 3.65, 3.83 and 3.97.





## Figure S25. Scatter plot of the lignin (1508 cm^-1^) peak area ratio relative to that of holocellulose (895 cm^-1^) from ATR-FTIR and the initial water contact angle of corn stover, *Miscanthus* × *giganteus* stalks and wheat straw hydrothermally pretreated at log *R_0_* = 3.65, 3.83 and 3.97.





## Figure S26. Scatter plot of the wax (2918 cm^-1^) peak area ratio relative to that of holocellulose (895 cm^-1^) from ATR-FTIR and the initial water contact angle of corn stover, *Miscanthus* × *giganteus* stalks and wheat straw hydrothermally pretreated at log *R_0_* = 3.65, 3.83 and 3.97.





## Figure S27. Scatter plot of cellulose (glucan) content from composition analysis and the initial water contact angle of corn stover, *Miscanthus* × *giganteus* stalks and wheat straw hydrothermally pretreated at log *R_0_* = 3.65, 3.83 and 3.97.





## Figure S28. Scatter plot of hemicellulose (arabino-/xylan) content from composition analysis and the initial water contact angle of corn stover, *Miscanthus* × *giganteus* stalks and wheat straw hydrothermally pretreated at log *R_0_* = 3.65, 3.83 and 3.97.





## Figure S29. Scatter plot of lignin content from composition analysis and the initial water contact angle of corn stover, *Miscanthus* × *giganteus* stalks and wheat straw hydrothermally pretreated at log *R_0_* = 3.65, 3.83 and 3.97.





## Figure S30. Scatter plot of hemicellulose (arabinose and xylose) removal from composition analysis and the initial water contact angle of corn stover, *Miscanthus* × *giganteus* stalks and wheat straw hydrothermally pretreated at log *R_0_* = 3.65, 3.83 and 3.97.

# References

1. Boerjan W, Ralph J, Baucher M. Lignin biosynthesis. Annu Rev Plant Biol. 2003;54:519–46.

2. Ralph J, Lundquist K, Brunow G, Lu F, Kim H, Schatz PF, Marita JM, Hatfield RD, Ralph SA, Christensen JH, Boerjan W. Lignins: natural polymers from oxidative coupling of 4-hydroxyphenyl- propanoids. Phytochem Rev. 2004;3:29–60.
